# Supplementary material for: Small molecule activators of the mitochondrial protease ClpP induce senescence in triple-negative breast cancer cells and sensitize cells to the Bcl-2 inhibitor venetoclax
Source: Cell Death Dis. 2026 Jun 29;17(1):803. doi: 10.1038/s41419-026-09061-w (PMC13578376; doi:10.1038/s41419-026-09061-w)
Supplement: Supplementary file 1 [file 41419_2026_9061_MOESM2_ESM.pdf]

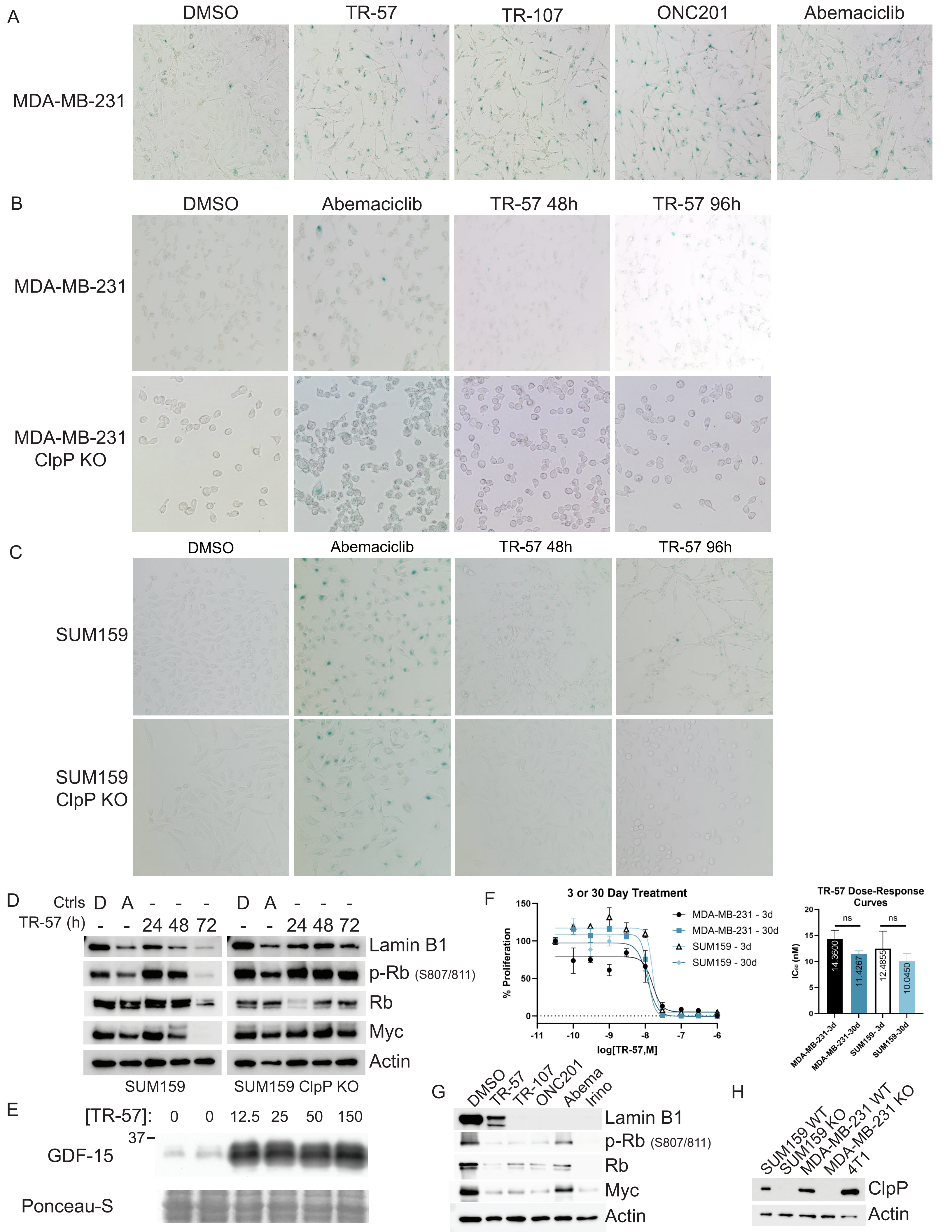

A
DMSO
TR-57
TR-107
ONC201
Abemaciclib
MDA-MB-231
B
DMSO
Abemaciclib
TR-57 48h
TR-57 96h
MDA-MB-231
MDA-MB-231 ClpP KO
C
DMSO
Abemaciclib
TR-57 48h
TR-57 96h
SUM159
SUM159 ClpP KO
D
Ctrls D A - - - D A - - -
TR-57 (h) - - 24 48 72 - - 24 48 72
Lamin B1
p-Rb (S807/811)
Rb
Myc
Actin
SUM159
SUM159 ClpP KO
E
[TR-57]: 0 0 12.5 25 50 150
37 –
GDF-15
Ponceau-S
F
3 or 30 Day Treatment
% Proliferation
log[TR-57,M]
MDA-MB-231 - 3d
MDA-MB-231 - 30d
SUM159 - 3d
SUM159 - 30d
TR-57 Dose-Response Curves
IC₅₀ (nM)
ns
ns
14.3600
11.4267
12.4855
10.0450
MDA-MB-231-3d
MDA-MB-231-30d
SUM159- 3d
SUM159-30d
G
DMSO
TR-57
TR-107
ONC201
Abema
Irino
Lamin B1
p-Rb (S807/811)
Rb
Myc
Actin
H
SUM159 WT
SUM159 KO
MDA-MB-231 WT
MDA-MB-231 KO
4T1
ClpP
Actin
