# Supplementary material for: Small molecule activators of the mitochondrial protease ClpP induce senescence in triple-negative breast cancer cells and sensitize cells to the Bcl-2 inhibitor venetoclax
Source: Cell Death Dis. 2026 Jun 29;17(1):803. doi: 10.1038/s41419-026-09061-w (PMC13578376; doi:10.1038/s41419-026-09061-w)
Supplement: Supplementary file 2 [file 41419_2026_9061_MOESM3_ESM.pdf]

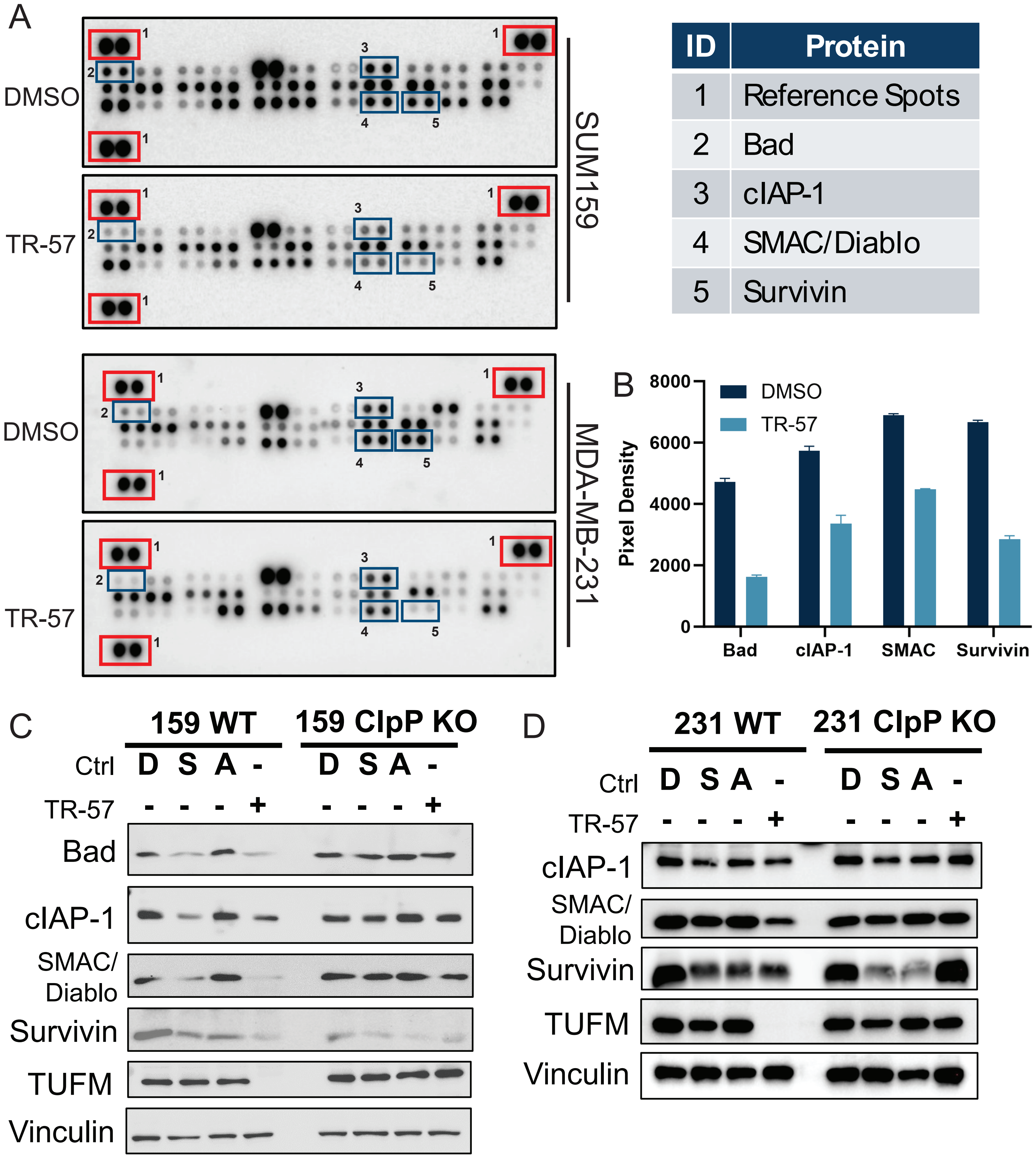

A
DMSO
TR-57
SUM159
DMSO
TR-57
MDA-MB-231
1
2
3
4
5
ID
Protein
1 Reference Spots
2 Bad
3 cIAP-1
4 SMAC/Diablo
5 Survivin
B
Pixel Density
8000
6000
4000
2000
0
DMSO
TR-57
Bad
cIAP-1
SMAC
Survivin
C
159 WT
159 ClpP KO
Ctrl
D S A -
TR-57
- - - +
Bad
cIAP-1
SMAC/ Diablo
Survivin
TUFM
Vinculin
D
231 WT
231 ClpP KO
Ctrl
D S A -
TR-57
- - - +
cIAP-1
SMAC/ Diablo
Survivin
TUFM
Vinculin
