# Supplementary material for: Small molecule activators of the mitochondrial protease ClpP induce senescence in triple-negative breast cancer cells and sensitize cells to the Bcl-2 inhibitor venetoclax
Source: Cell Death Dis. 2026 Jun 29;17(1):803. doi: 10.1038/s41419-026-09061-w (PMC13578376; doi:10.1038/s41419-026-09061-w)
Supplement: Supplementary file 3 [file 41419_2026_9061_MOESM4_ESM.pdf]

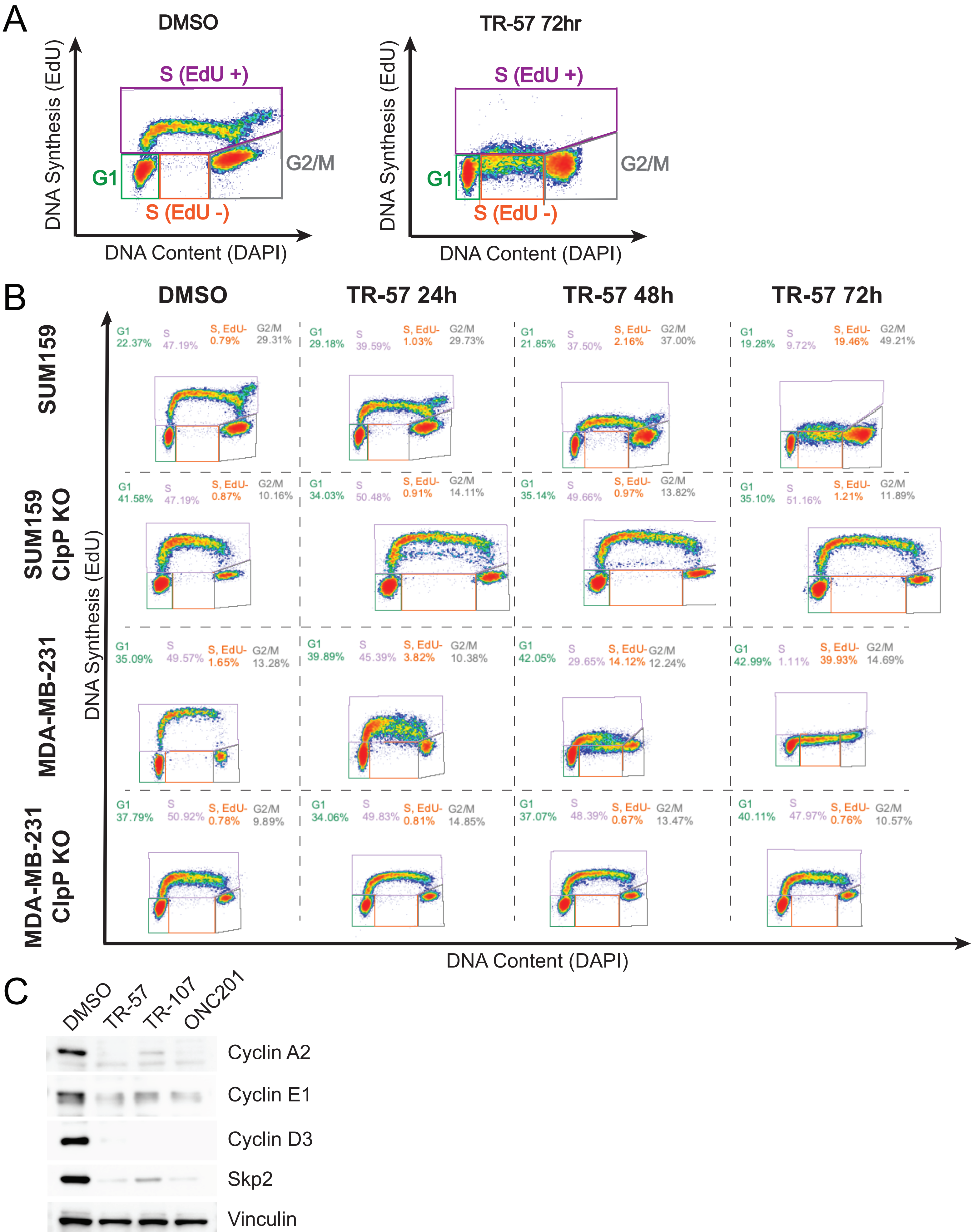
A
DMSO
TR-57 72hr
S (EdU +)
G1
G2/M
S (EdU -)
DNA Synthesis (EdU)
DNA Content (DAPI)
B
DMSO
TR-57 24h
TR-57 48h
TR-57 72h
SUM159
SUM159 ClpP KO
MDA-MB-231
MDA-MB-231 ClpP KO
G1 22.37% S 47.19% S, EdU- 0.79% G2/M 29.31%
G1 29.18% S 39.59% S, EdU- 1.03% G2/M 29.73%
G1 21.85% S 37.50% S, EdU- 2.16% G2/M 37.00%
G1 19.28% S 9.72% S, EdU- 19.46% G2/M 49.21%
G1 41.58% S 47.19% S, EdU- 0.87% G2/M 10.16%
G1 34.03% S 50.48% S, EdU- 0.91% G2/M 14.11%
G1 35.14% S 49.66% S, EdU- 0.97% G2/M 13.82%
G1 35.10% S 51.16% S, EdU- 1.21% G2/M 11.89%
G1 35.09% S 49.57% S, EdU- 1.65% G2/M 13.28%
G1 39.89% S 45.39% S, EdU- 3.82% G2/M 10.38%
G1 42.05% S 29.65% S, EdU- 14.12% G2/M 12.24%
G1 42.99% S 1.11% S, EdU- 39.93% G2/M 14.69%
G1 37.79% S 50.92% S, EdU- 0.78% G2/M 9.89%
G1 34.06% S 49.83% S, EdU- 0.81% G2/M 14.85%
G1 37.07% S 48.39% S, EdU- 0.67% G2/M 13.47%
G1 40.11% S 47.97% S, EdU- 0.76% G2/M 10.57%
DNA Synthesis (EdU)
DNA Content (DAPI)
C
DMSO
TR-57
TR-107
ONC201
Cyclin A2
Cyclin E1
Cyclin D3
Skp2
Vinculin
