# Supplementary material for: Small molecule activators of the mitochondrial protease ClpP induce senescence in triple-negative breast cancer cells and sensitize cells to the Bcl-2 inhibitor venetoclax
Source: Cell Death Dis. 2026 Jun 29;17(1):803. doi: 10.1038/s41419-026-09061-w (PMC13578376; doi:10.1038/s41419-026-09061-w)
Supplement: Supplementary file 4 [file 41419_2026_9061_MOESM5_ESM.pdf]

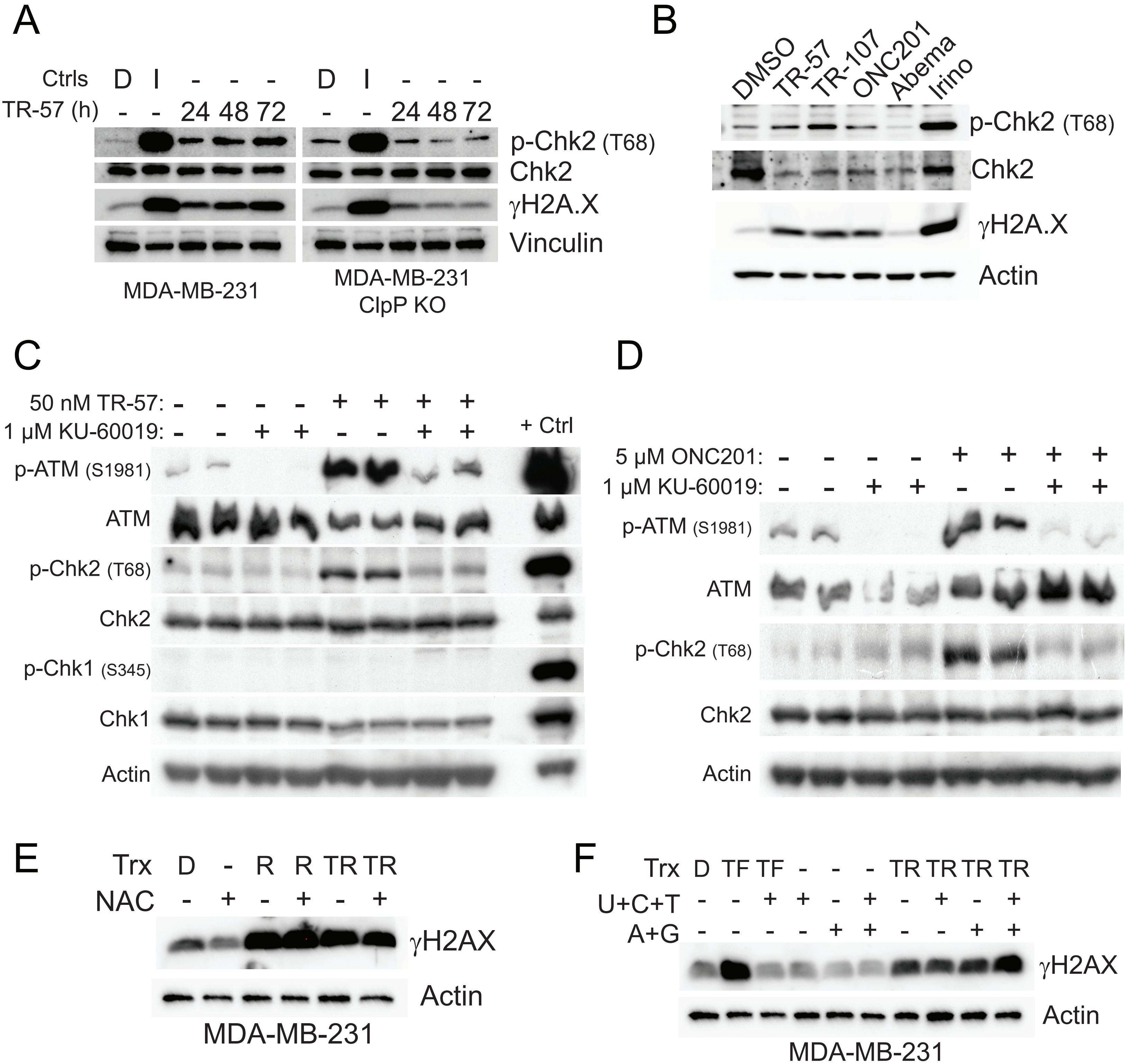
A
Ctrls D I - - - D I - - -
TR-57 (h) - - 24 48 72 - - 24 48 72
p-Chk2 (T68)
Chk2
γH2A.X
Vinculin
MDA-MB-231
MDA-MB-231 ClpP KO
B
DMSO
TR-57
TR-107
ONC201
Abema
Irino
p-Chk2 (T68)
Chk2
γH2A.X
Actin
C
50 nM TR-57: - - - - + + + +
1 μM KU-60019: - - + + - - + +
+ Ctrl
p-ATM (S1981)
ATM
p-Chk2 (T68)
Chk2
p-Chk1 (S345)
Chk1
Actin
D
5 μM ONC201: - - - - + + + +
1 μM KU-60019: - - + + - - + +
p-ATM (S1981)
ATM
p-Chk2 (T68)
Chk2
Actin
E
Trx D - R R TR TR
NAC - + - + - +
γH2AX
Actin
MDA-MB-231
F
Trx D TF TF - - - TR TR TR TR
U+C+T - - + + - + - + - +
A+G - - - - + + - - + +
γH2AX
Actin
MDA-MB-231
