# Supplementary material for: Small molecule activators of the mitochondrial protease ClpP induce senescence in triple-negative breast cancer cells and sensitize cells to the Bcl-2 inhibitor venetoclax
Source: Cell Death Dis. 2026 Jun 29;17(1):803. doi: 10.1038/s41419-026-09061-w (PMC13578376; doi:10.1038/s41419-026-09061-w)
Supplement: Supplementary file 5 [file 41419_2026_9061_MOESM6_ESM.pdf]

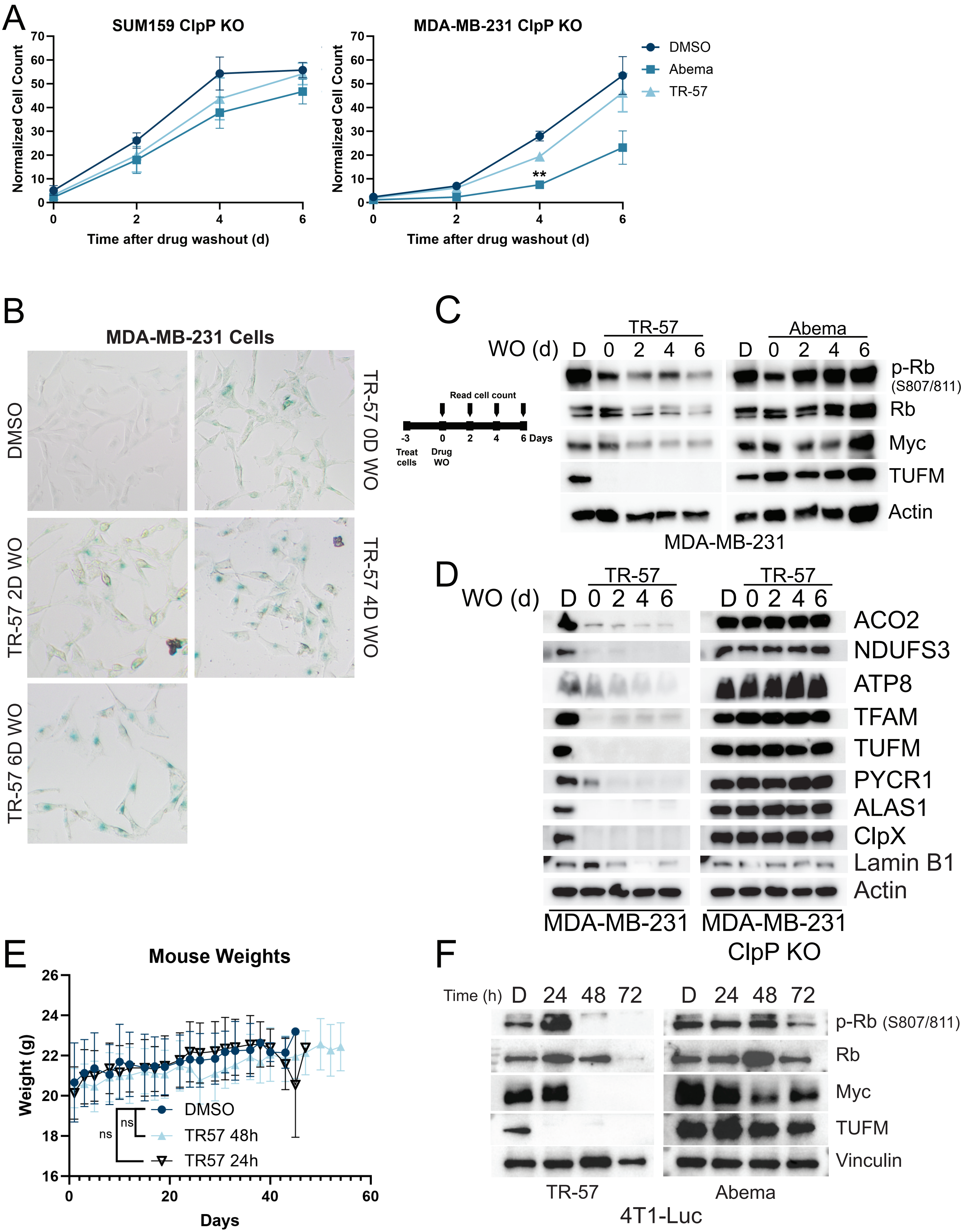

A
SUM159 ClpP KO
MDA-MB-231 ClpP KO
Normalized Cell Count
Time after drug washout (d)
DMSO
Abema
TR-57
**
B
MDA-MB-231 Cells
DMSO
TR-57 0D WO
TR-57 2D WO
TR-57 4D WO
TR-57 6D WO
C
Read cell count
-3 Treat cells
0 Drug WO
2
4
6 Days
WO (d)
TR-57
Abema
D 0 2 4 6
p-Rb (S807/811)
Rb
Myc
TUFM
Actin
MDA-MB-231
D
WO (d)
TR-57
ACO2
NDUFS3
ATP8
TFAM
TUFM
PYCR1
ALAS1
ClpX
Lamin B1
Actin
MDA-MB-231
MDA-MB-231 ClpP KO
E
Mouse Weights
Weight (g)
Days
DMSO
TR57 48h
TR57 24h
ns
F
Time (h) D 24 48 72
p-Rb (S807/811)
Rb
Myc
TUFM
Vinculin
TR-57
Abema
4T1-Luc
