# Supplementary material for: Small molecule activators of the mitochondrial protease ClpP induce senescence in triple-negative breast cancer cells and sensitize cells to the Bcl-2 inhibitor venetoclax
Source: Cell Death Dis. 2026 Jun 29;17(1):803. doi: 10.1038/s41419-026-09061-w (PMC13578376; doi:10.1038/s41419-026-09061-w)
Supplement: Supplementary file 6 [file 41419_2026_9061_MOESM7_ESM.pdf]

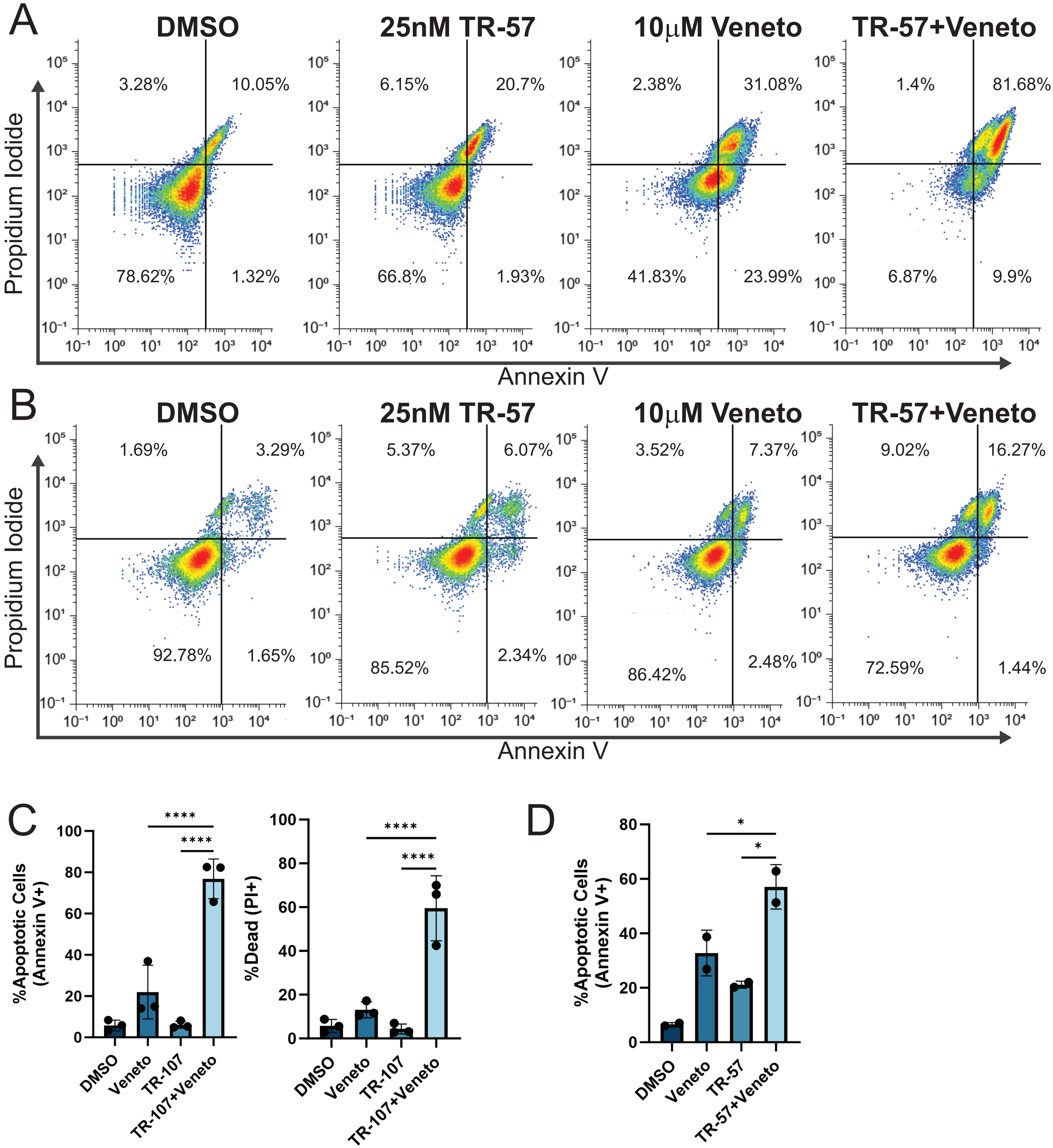
A
DMSO
25nM TR-57
10μM Veneto
TR-57+Veneto
Propidium Iodide
Annexin V
3.28%
10.05%
78.62%
1.32%
6.15%
20.7%
66.8%
1.93%
2.38%
31.08%
41.83%
23.99%
1.4%
81.68%
6.87%
9.9%
B
DMSO
25nM TR-57
10μM Veneto
TR-57+Veneto
1.69%
3.29%
92.78%
1.65%
5.37%
6.07%
85.52%
2.34%
3.52%
7.37%
86.42%
2.48%
9.02%
16.27%
72.59%
1.44%
C
%Apoptotic Cells (Annexin V+)
%Dead (PI+)
DMSO
Veneto
TR-107
TR-107+Veneto
****
D
%Apoptotic Cells (Annexin V+)
DMSO
Veneto
TR-57
TR-57+Veneto
*
