# Supplementary material for: Small molecule activators of the mitochondrial protease ClpP induce senescence in triple-negative breast cancer cells and sensitize cells to the Bcl-2 inhibitor venetoclax
Source: Cell Death Dis. 2026 Jun 29;17(1):803. doi: 10.1038/s41419-026-09061-w (PMC13578376; doi:10.1038/s41419-026-09061-w)

Figure 1E

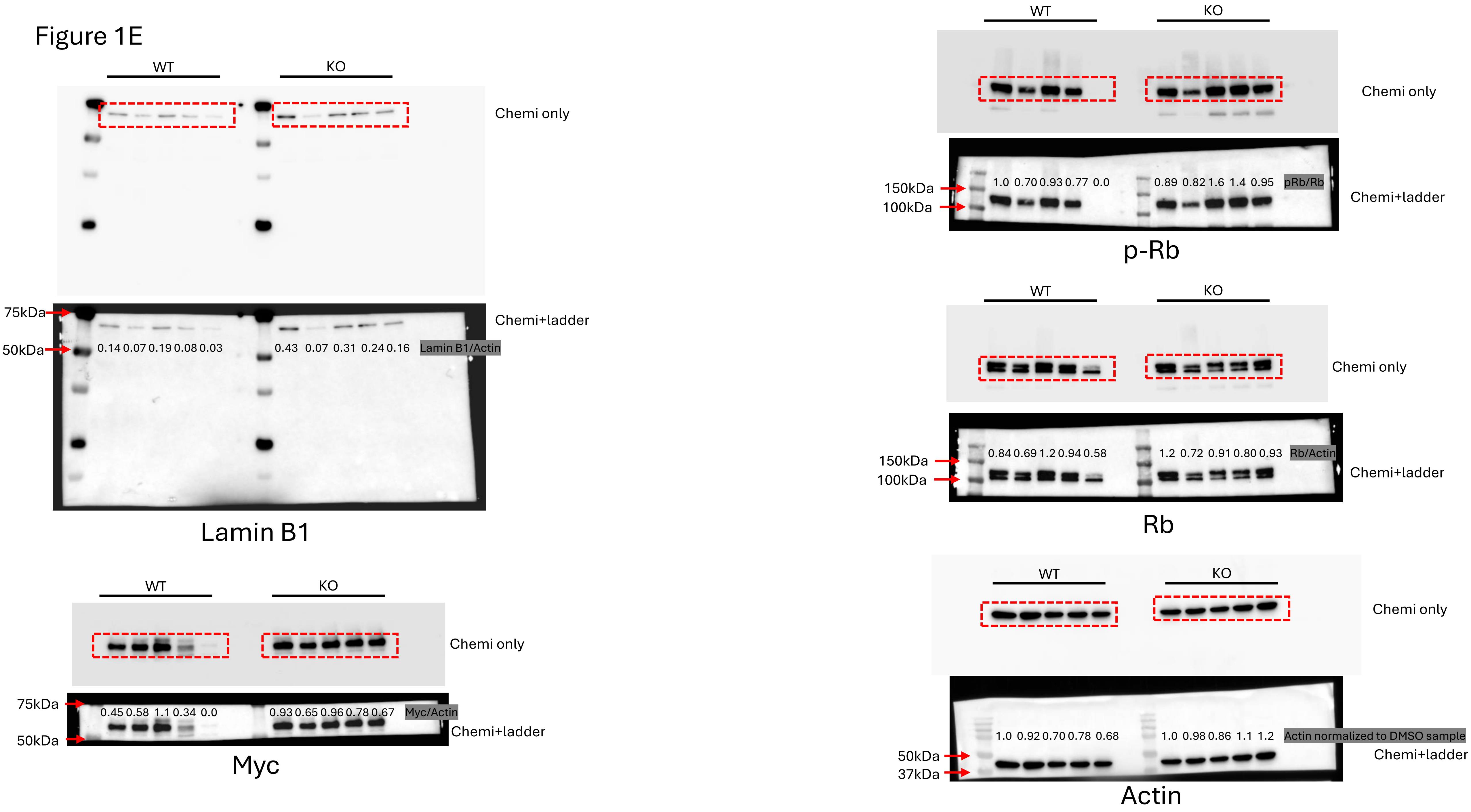

WT
KO
Chemi only
75kDa
50kDa
0.14 0.07 0.19 0.08 0.03
0.43 0.07 0.31 0.24 0.16
Lamin B1/Actin
Chemi+ladder
Lamin B1
WT
KO
Chemi only
75kDa
50kDa
0.45 0.58 1.1 0.34 0.0
0.93 0.65 0.96 0.78 0.67
Myc/Actin
Chemi+ladder
Myc
WT
KO
Chemi only
150kDa
100kDa
1.0 0.70 0.93 0.77 0.0
0.89 0.82 1.6 1.4 0.95
pRb/Rb
Chemi+ladder
p-Rb
WT
KO
Chemi only
150kDa
100kDa
0.84 0.69 1.2 0.94 0.58
1.2 0.72 0.91 0.80 0.93
Rb/Actin
Chemi+ladder
Rb
WT
KO
Chemi only
50kDa
37kDa
1.0 0.92 0.70 0.78 0.68
1.0 0.98 0.86 1.1 1.2
Actin normalized to DMSO sample
Chemi+ladder
Actin

Supplementary Figure 1D

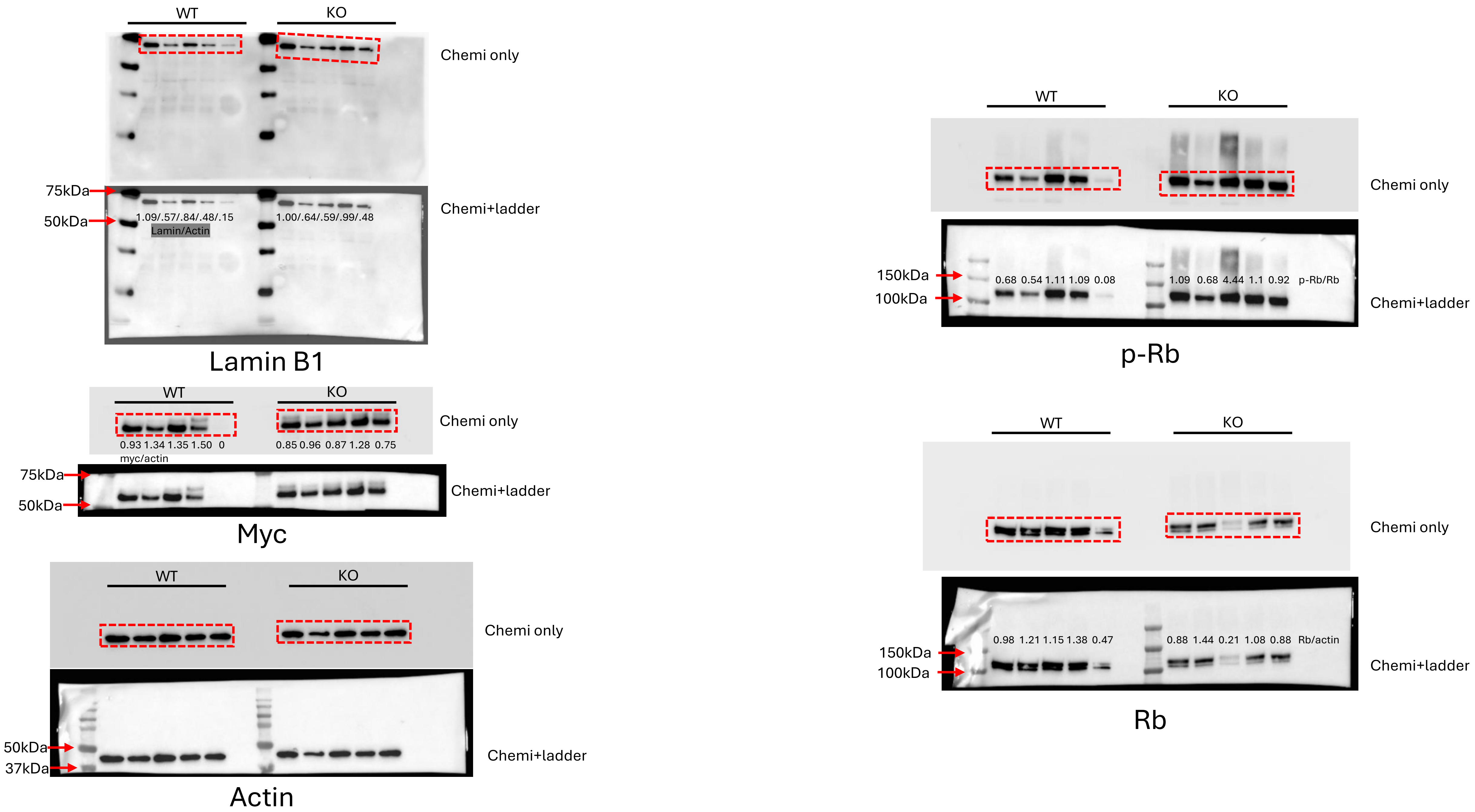

Supplementary Figure 1E, 1G

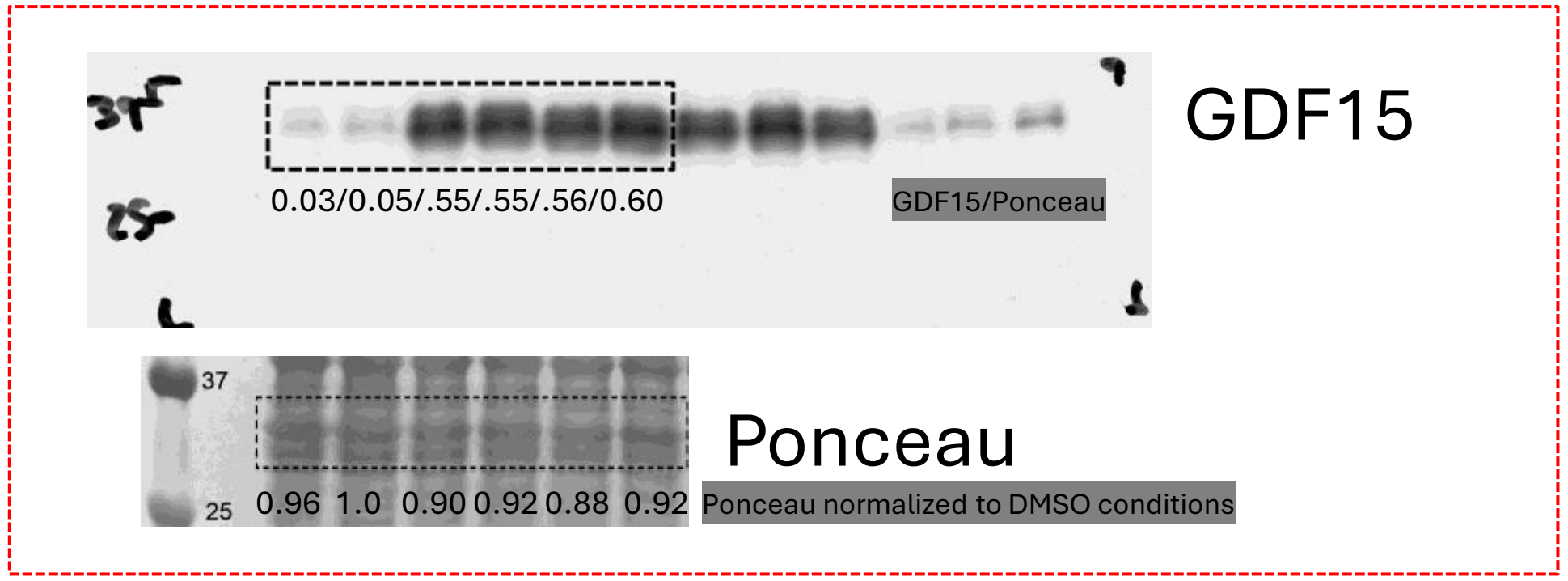

Supp Fig 1E

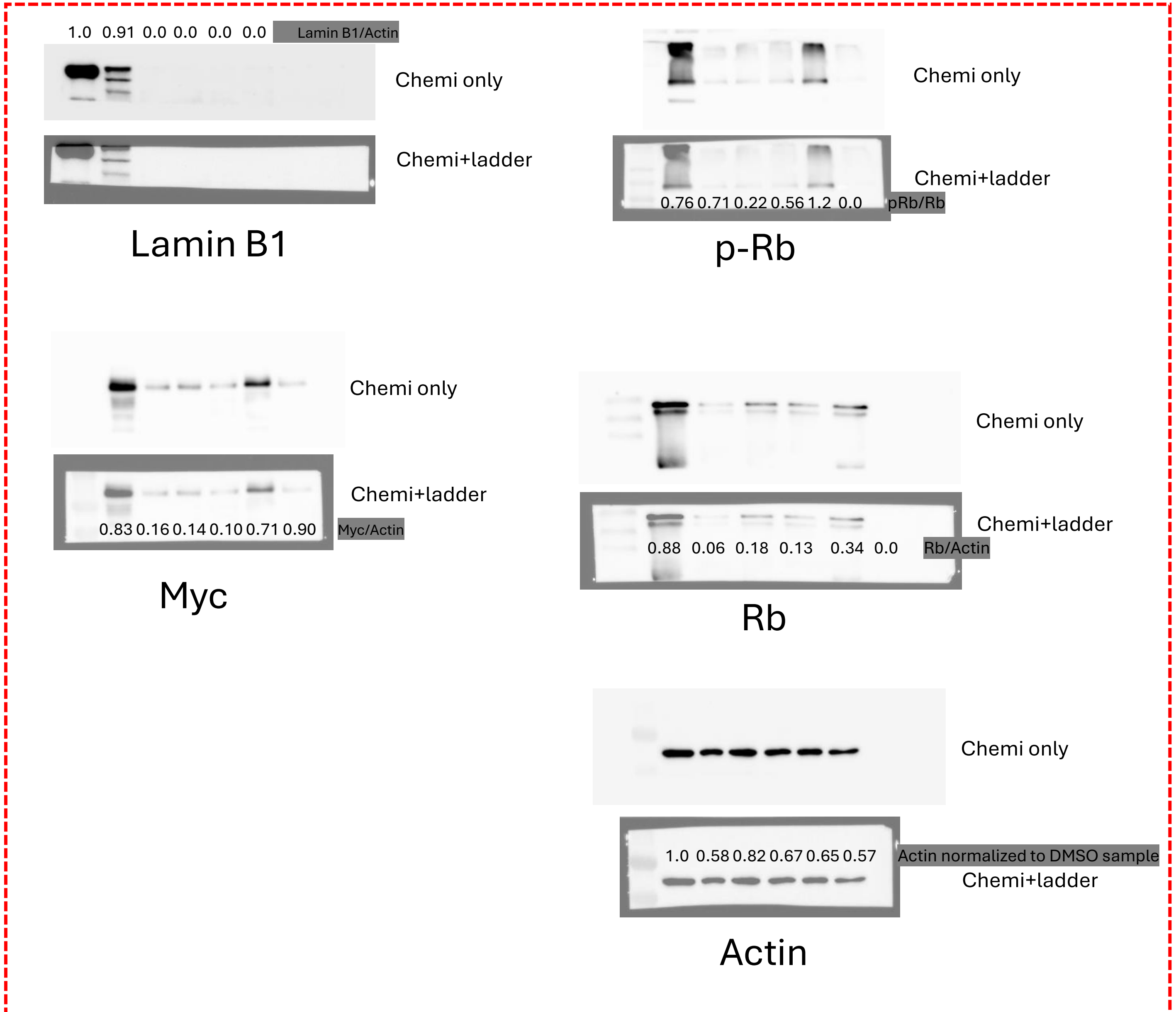

Supp Fig 1G

Supplementary Figure 2C

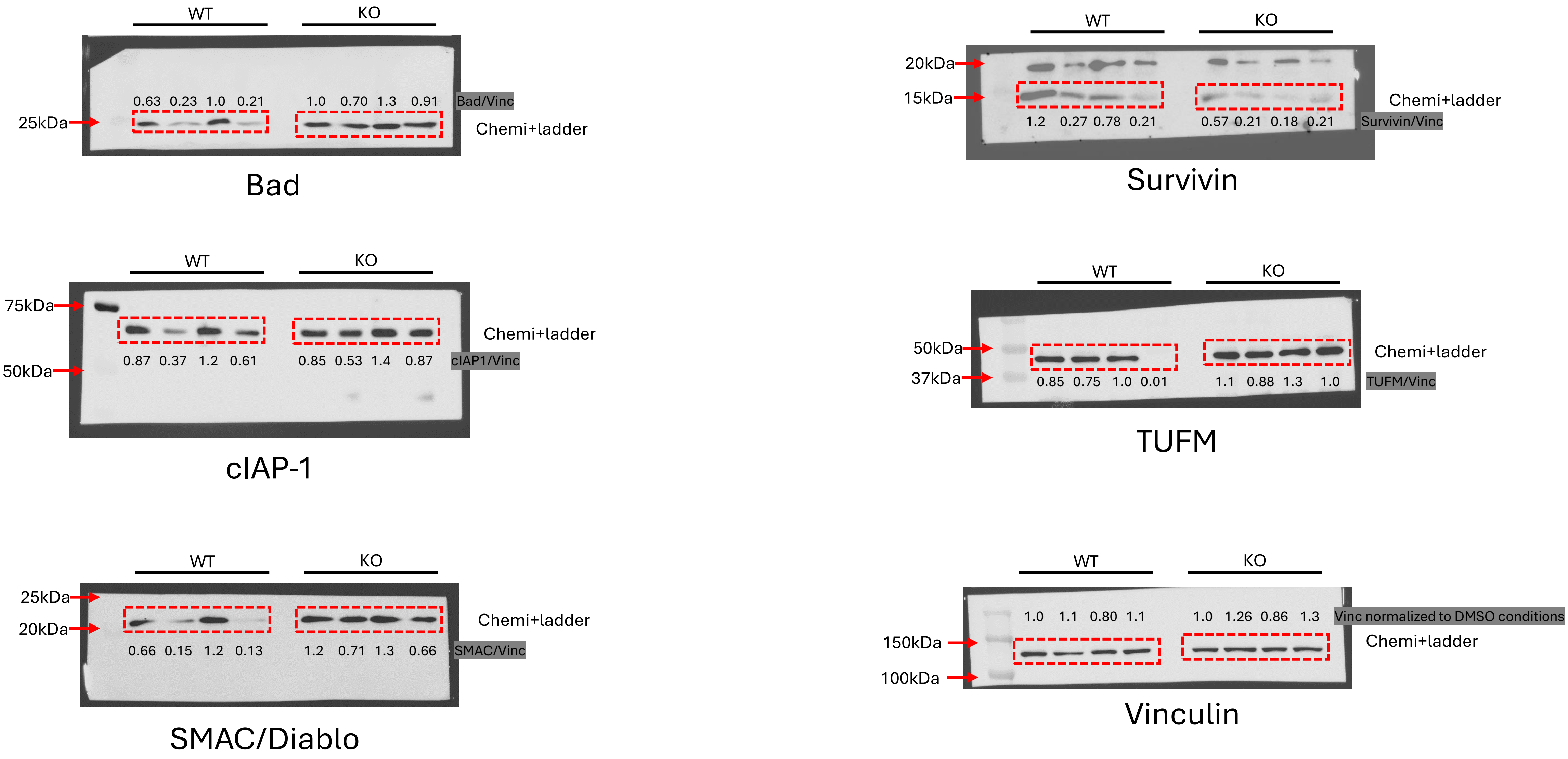

Supplementary Figure 2D

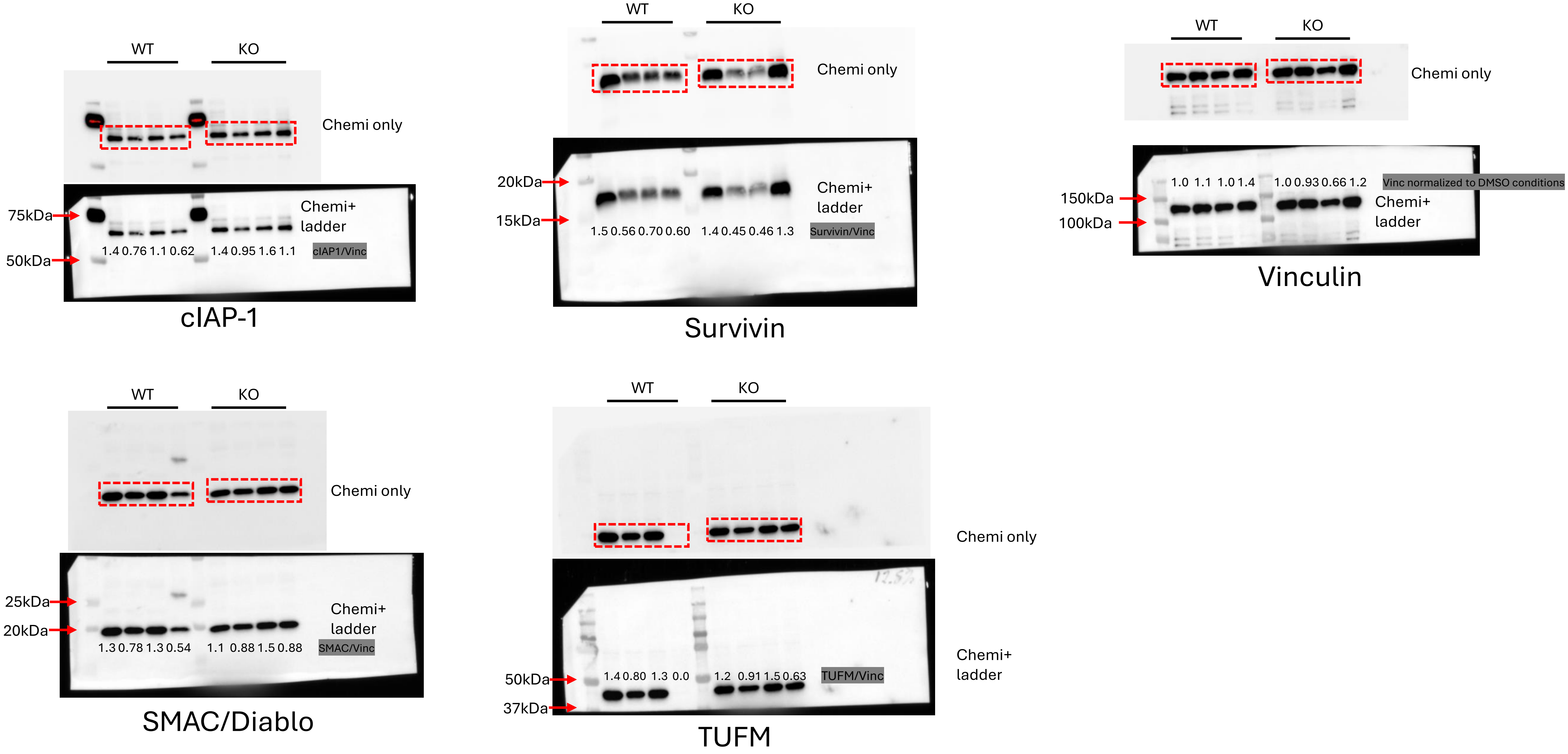

Figure 3D

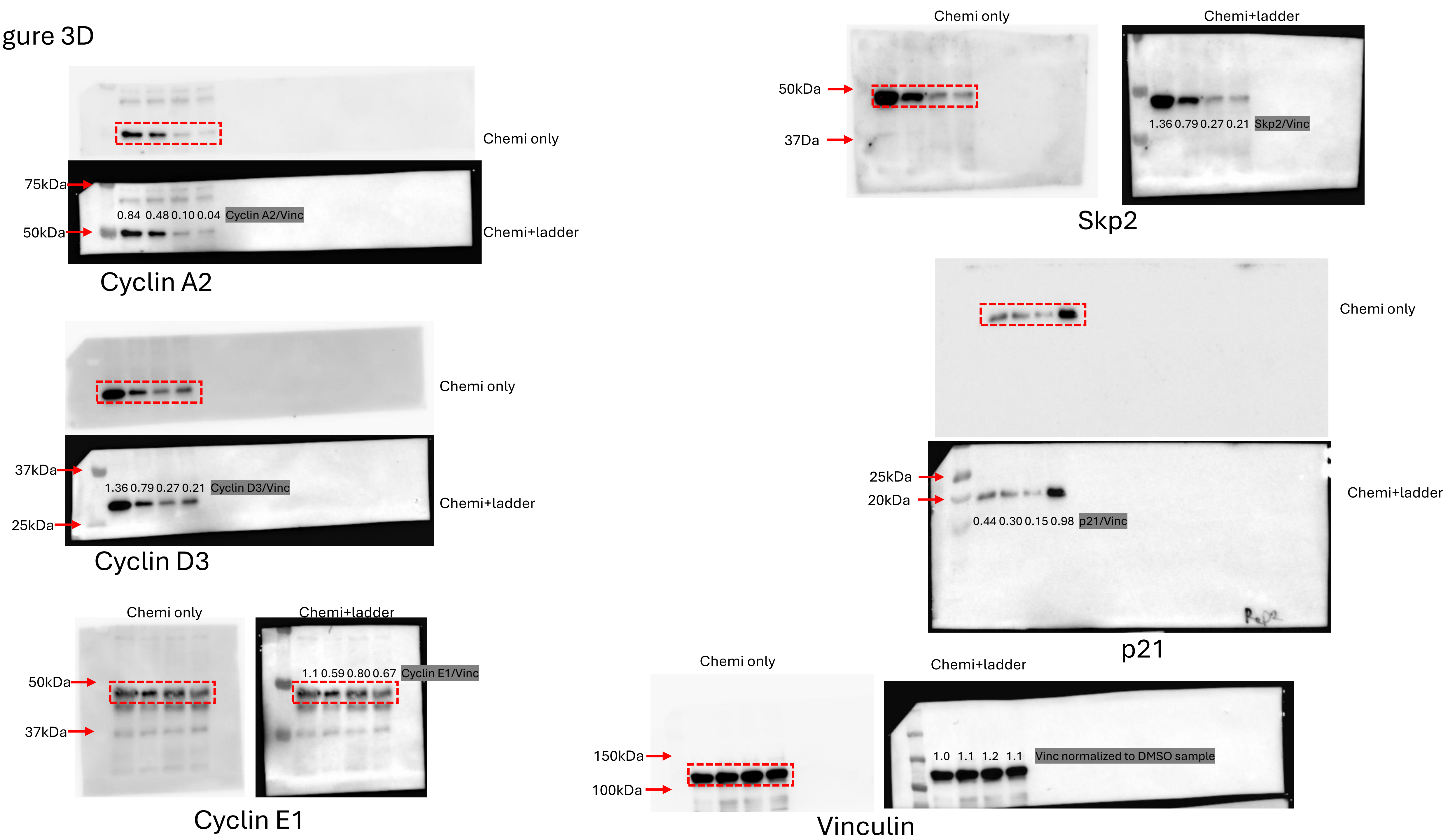

Chemi only
75kDa
50kDa
0.84 0.48 0.10 0.04
Cyclin A2/Vinc
Chemi+ladder
Cyclin A2
Chemi only
37kDa
25kDa
1.36 0.79 0.27 0.21
Cyclin D3/Vinc
Chemi+ladder
Cyclin D3
Chemi only
Chemi+ladder
50kDa
37kDa
1.1 0.59 0.80 0.67
Cyclin E1/Vinc
Cyclin E1
Chemi only
Chemi+ladder
50kDa
37Da
1.36 0.79 0.27 0.21
Skp2/Vinc
Skp2
Chemi only
25kDa
20kDa
0.44 0.30 0.15 0.98
p21/Vinc
Chemi+ladder
p21
Chemi only
Chemi+ladder
150kDa
100kDa
1.0 1.1 1.2 1.1
Vinc normalized to DMSO sample
Vinculin

Supplementary Figure 3C

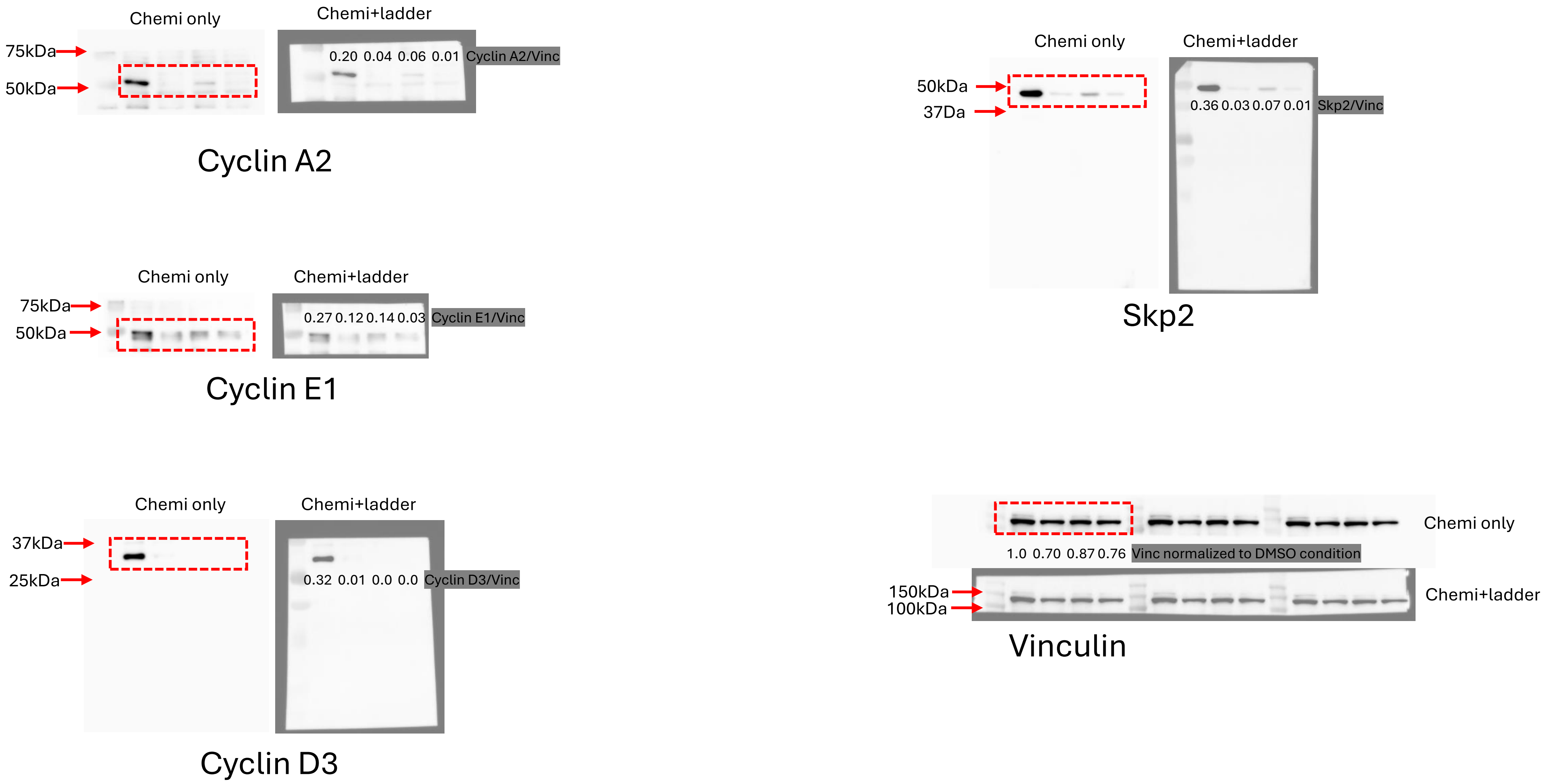

Figure 4A

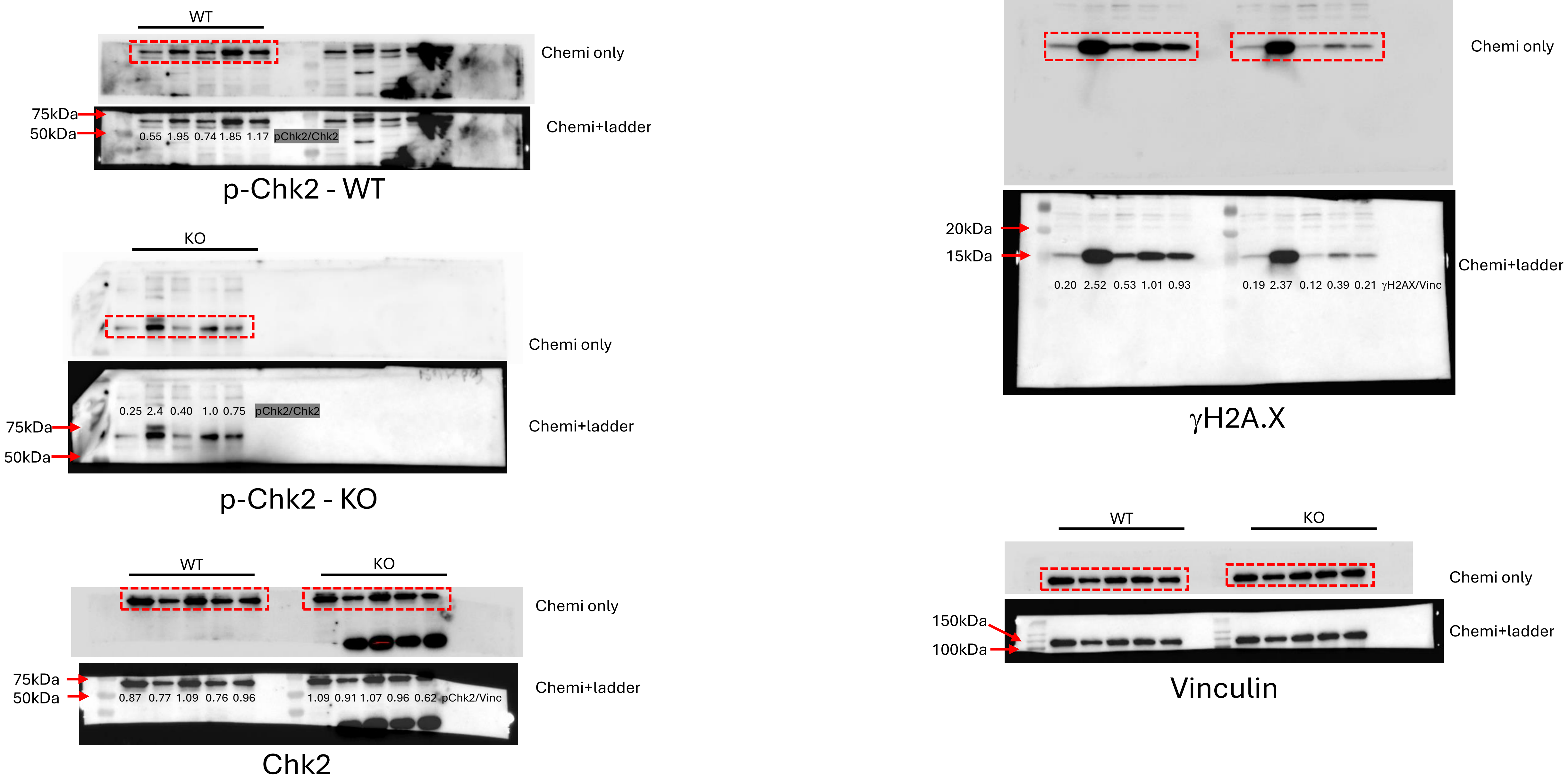

WT
Chemi only
75kDa
50kDa
0.55 1.95 0.74 1.85 1.17 pChk2/Chk2
Chemi+ladder
p-Chk2 - WT
KO
Chemi only
0.25 2.4 0.40 1.0 0.75 pChk2/Chk2
75kDa
50kDa
Chemi+ladder
p-Chk2 - KO
WT
KO
Chemi only
75kDa
50kDa
0.87 0.77 1.09 0.76 0.96
1.09 0.91 1.07 0.96 0.62 pChk2/Vinc
Chemi+ladder
Chk2
WT
KO
Chemi only
20kDa
15kDa
0.20 2.52 0.53 1.01 0.93
0.19 2.37 0.12 0.39 0.21 γH2AX/Vinc
Chemi+ladder
γH2A.X
WT
KO
Chemi only
150kDa
100kDa
Chemi+ladder
Vinculin

Supplementary Figure 4A

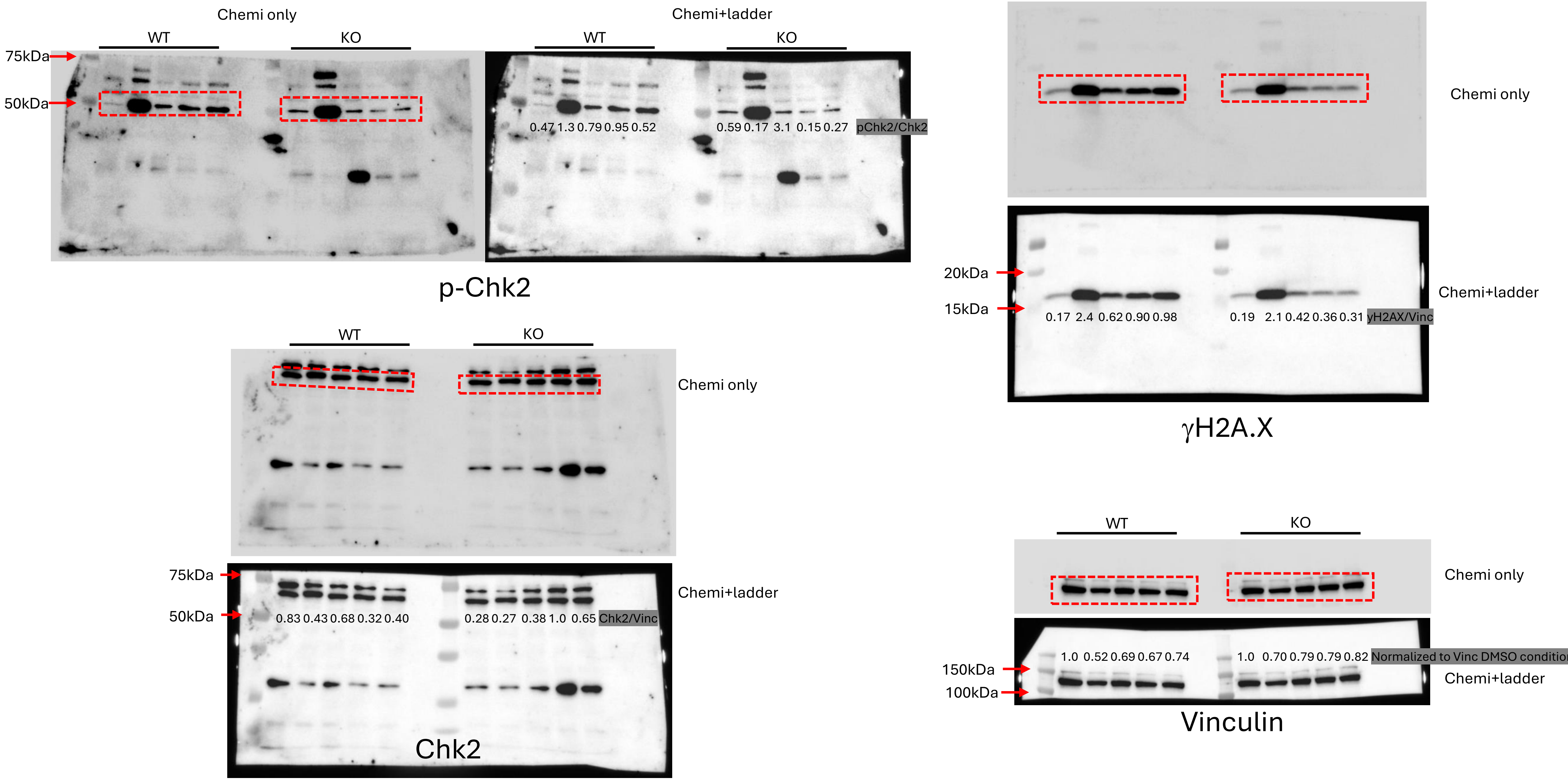

Supplementary Figure 4B

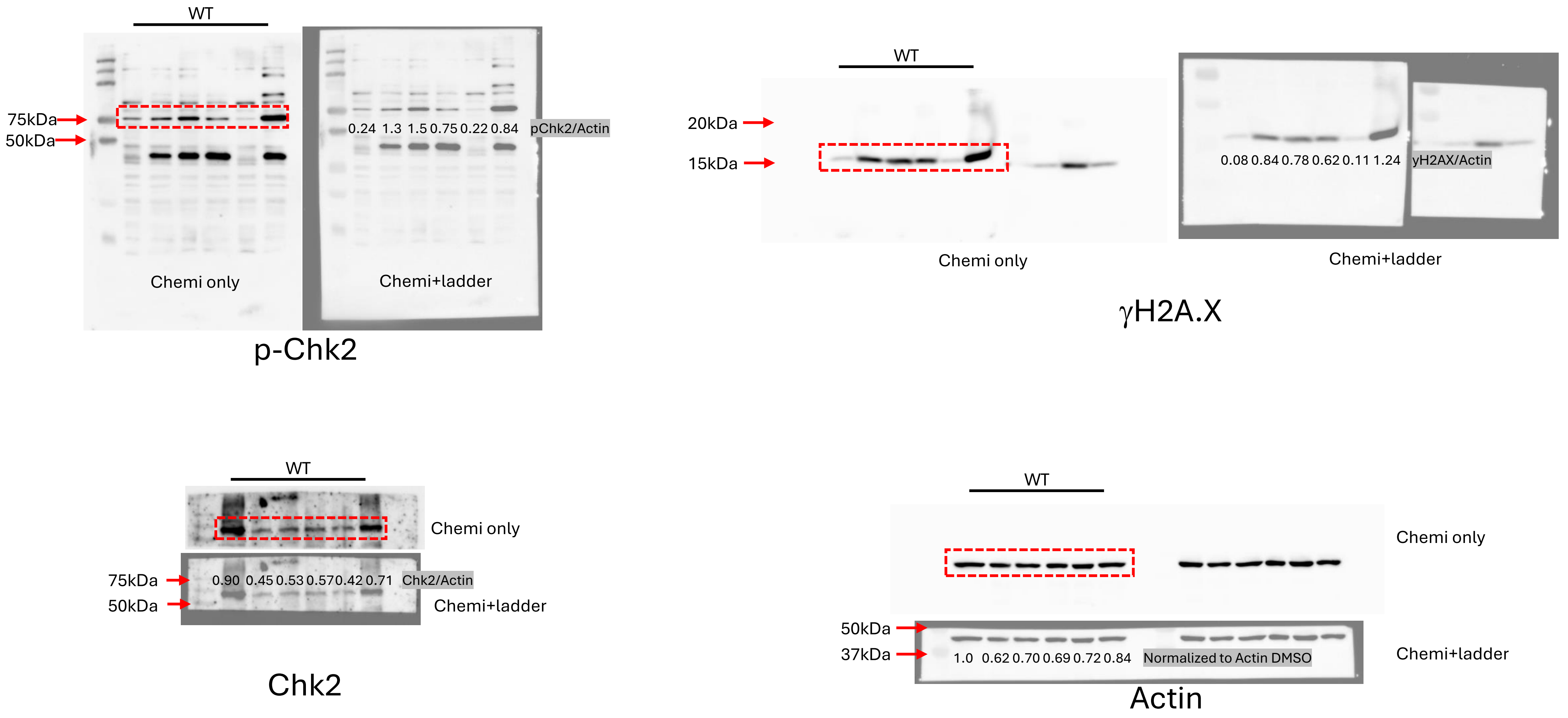

Supplementary Figure 4C

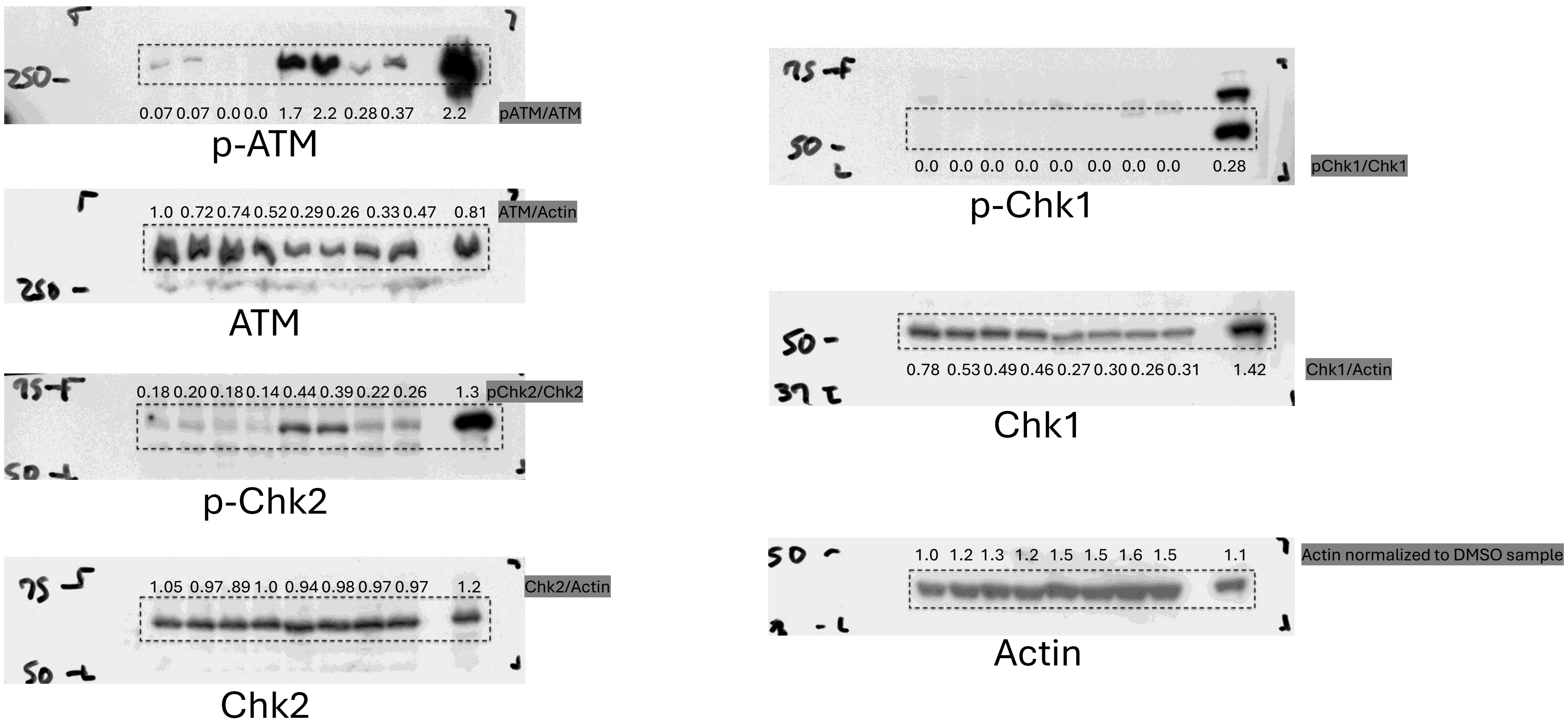

Supplementary Figure 4D

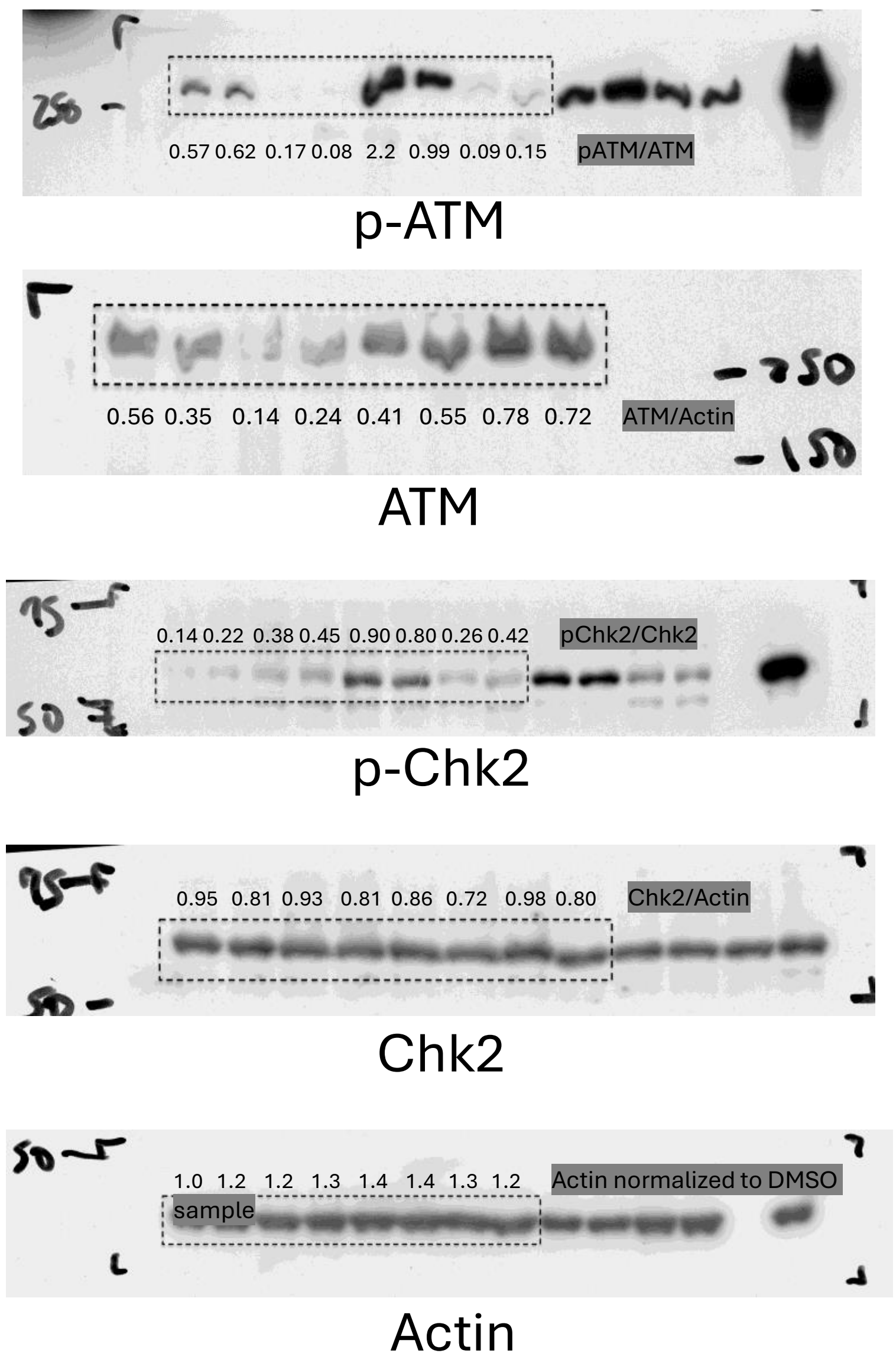

Supplementary Figure 4E, 4F

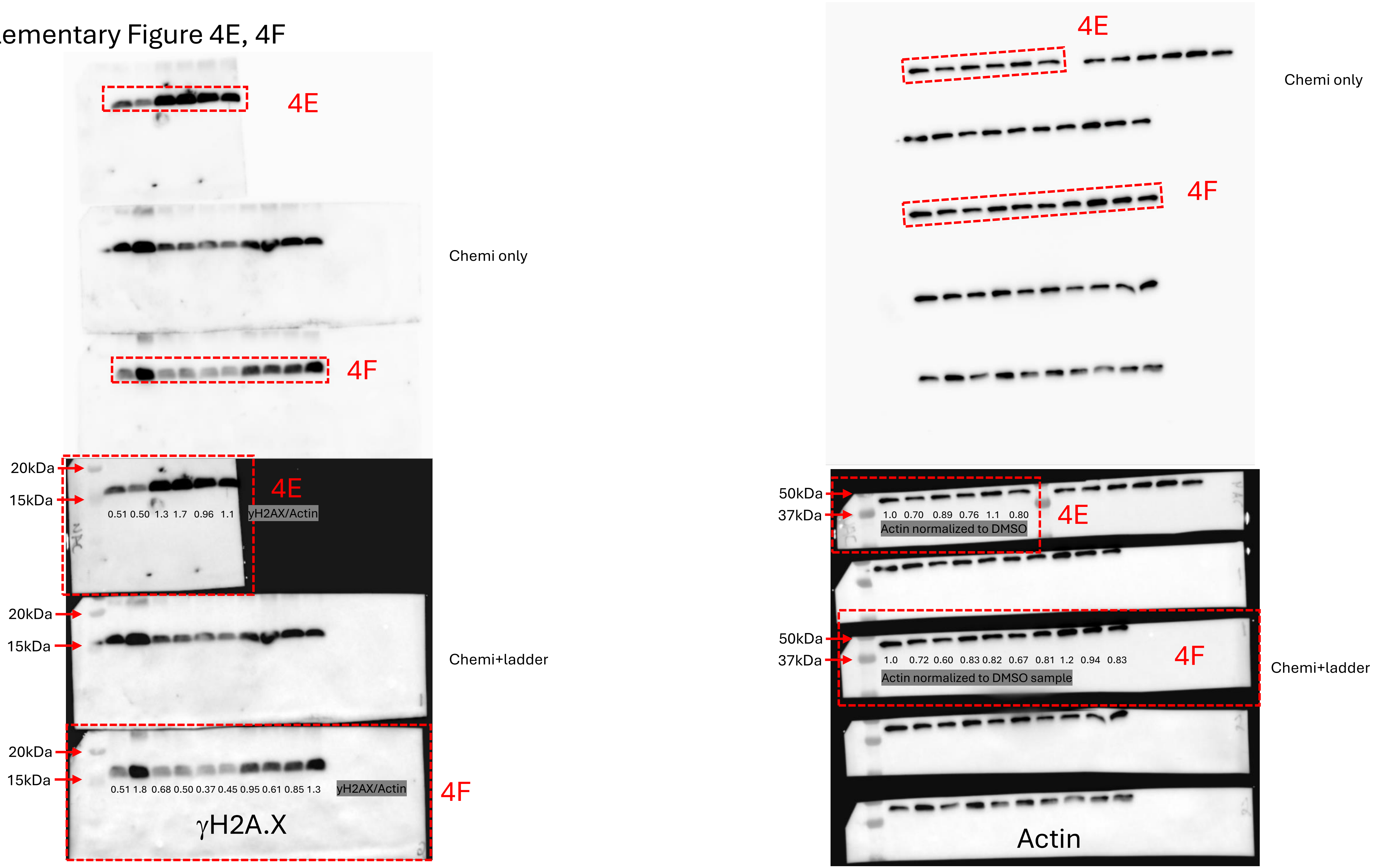

Figure 5C

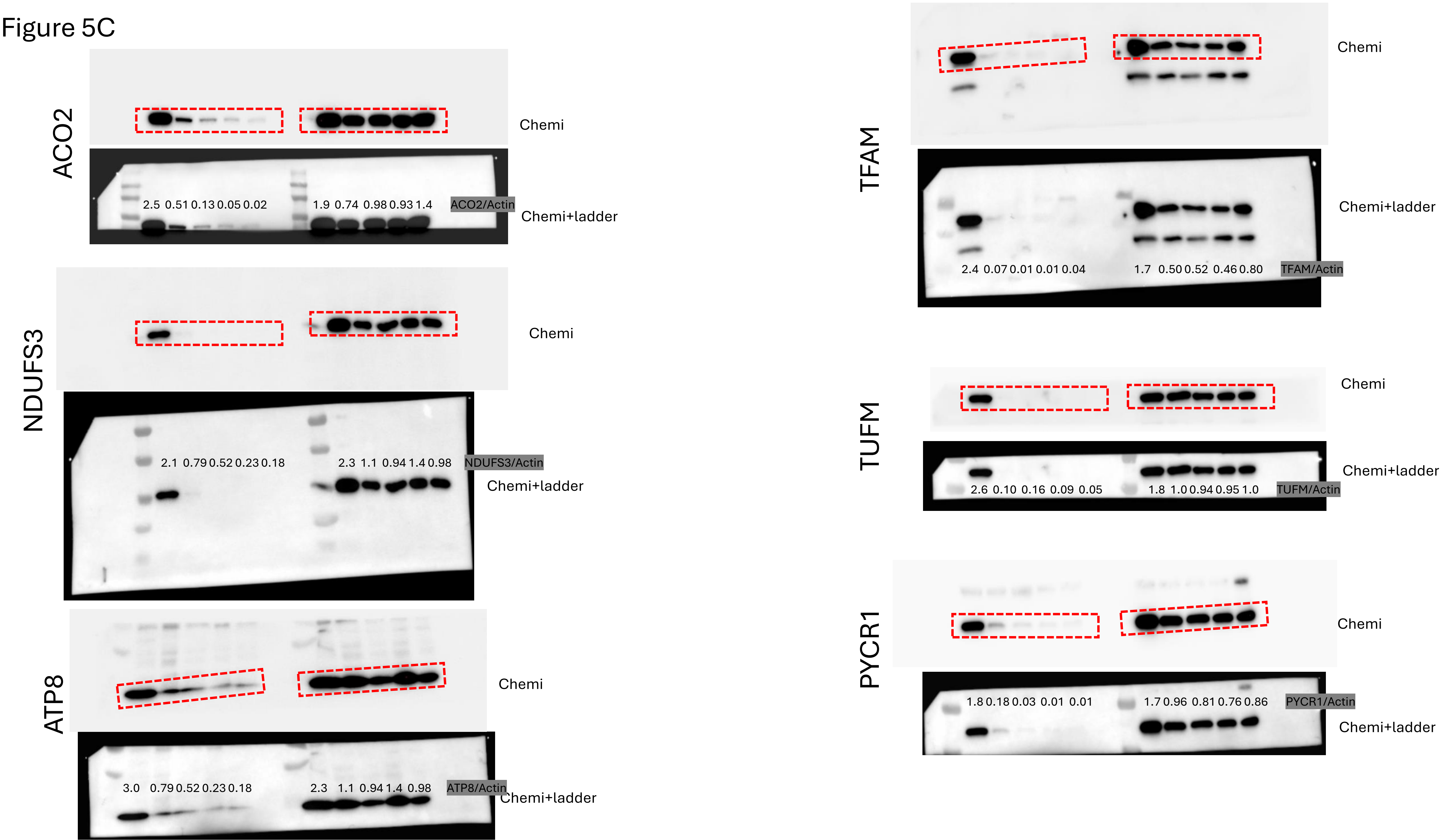
ACO2
Chemi
2.5 0.51 0.13 0.05 0.02
1.9 0.74 0.98 0.93 1.4
ACO2/Actin
Chemi+ladder
NDUFS3
Chemi
2.1 0.79 0.52 0.23 0.18
2.3 1.1 0.94 1.4 0.98
NDUFS3/Actin
Chemi+ladder
ATP8
Chemi
3.0 0.79 0.52 0.23 0.18
2.3 1.1 0.94 1.4 0.98
ATP8/Actin
Chemi+ladder
TFAM
Chemi
Chemi+ladder
2.4 0.07 0.01 0.01 0.04
1.7 0.50 0.52 0.46 0.80
TFAM/Actin
TUFM
Chemi
Chemi+ladder
2.6 0.10 0.16 0.09 0.05
1.8 1.0 0.94 0.95 1.0
TUFM/Actin
PYCR1
Chemi
1.8 0.18 0.03 0.01 0.01
1.7 0.96 0.81 0.76 0.86
PYCR1/Actin
Chemi+ladder

Figure 5C continued

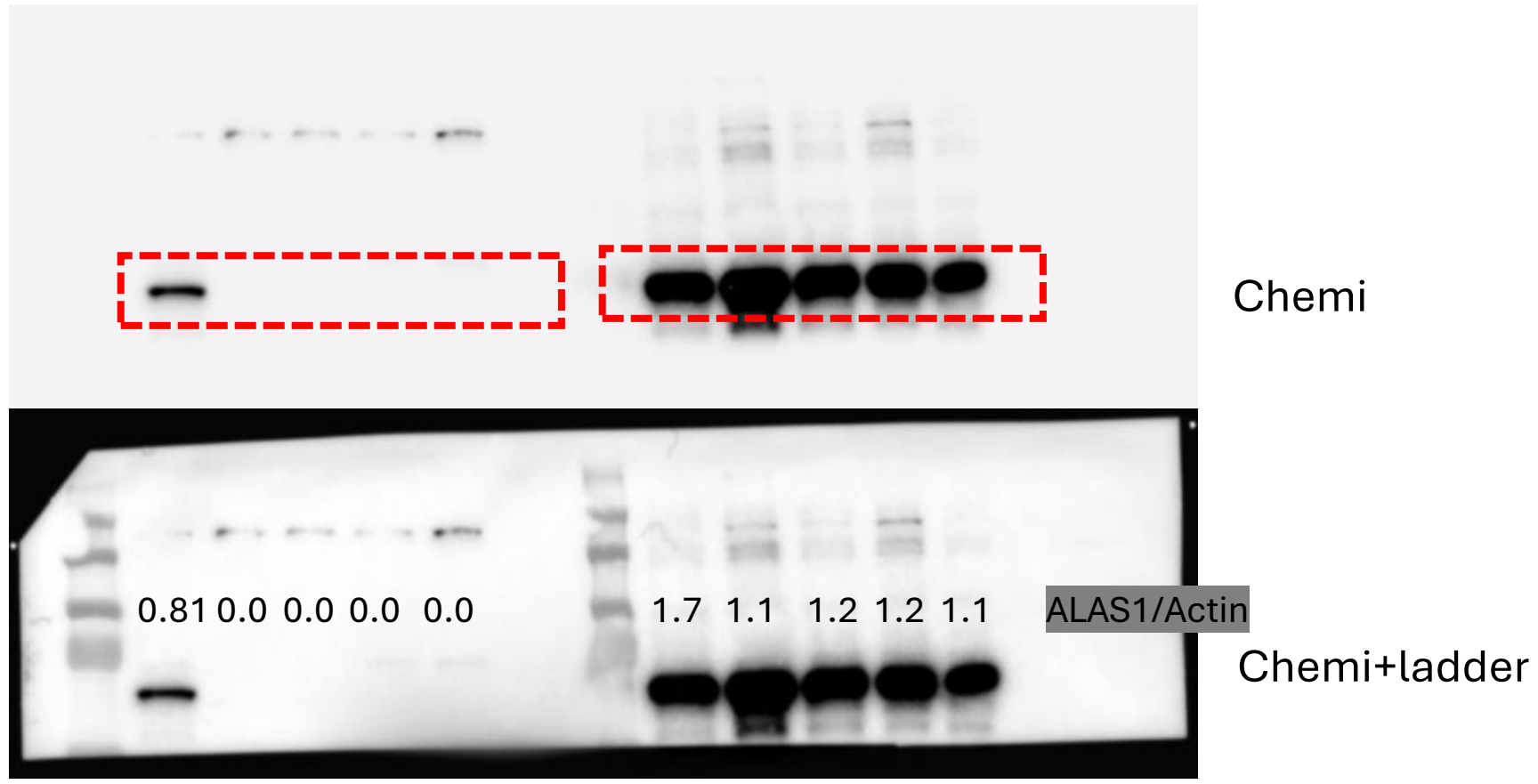

ALAS1

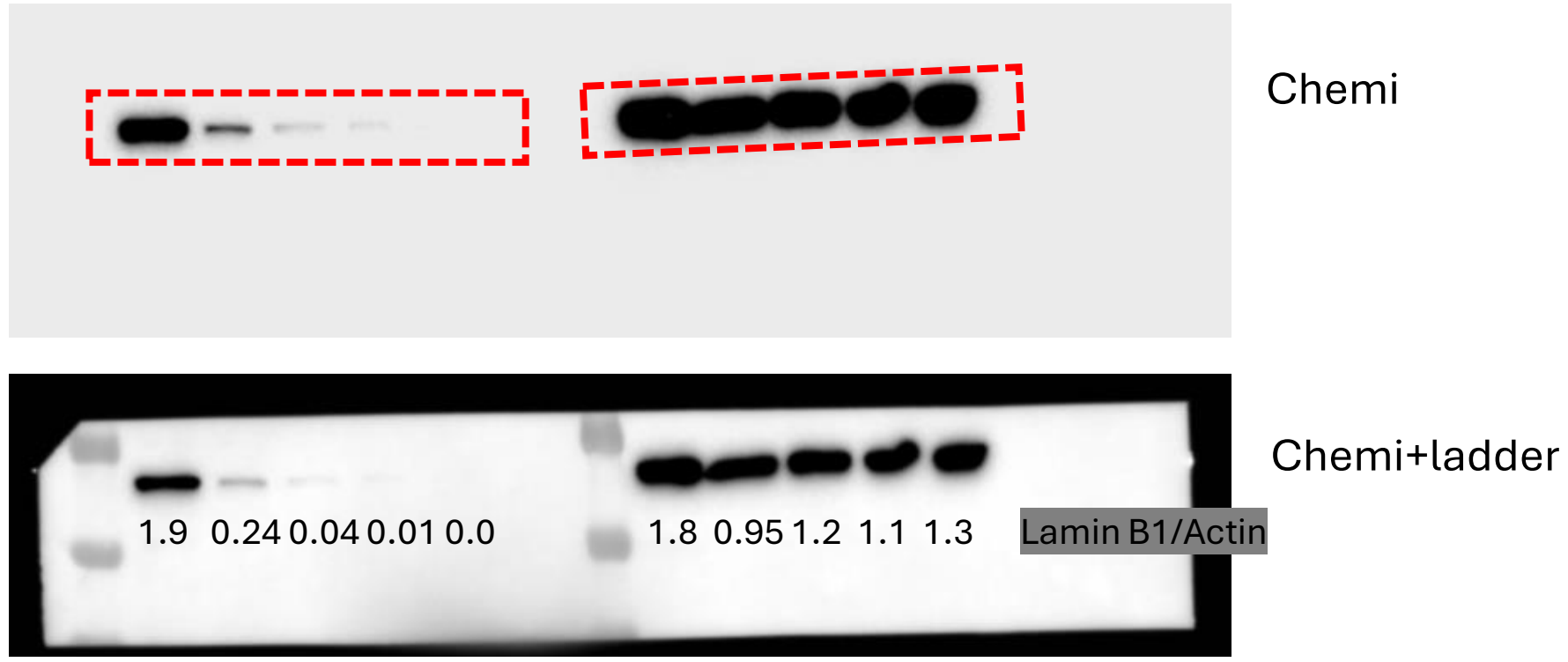

Lamin B1

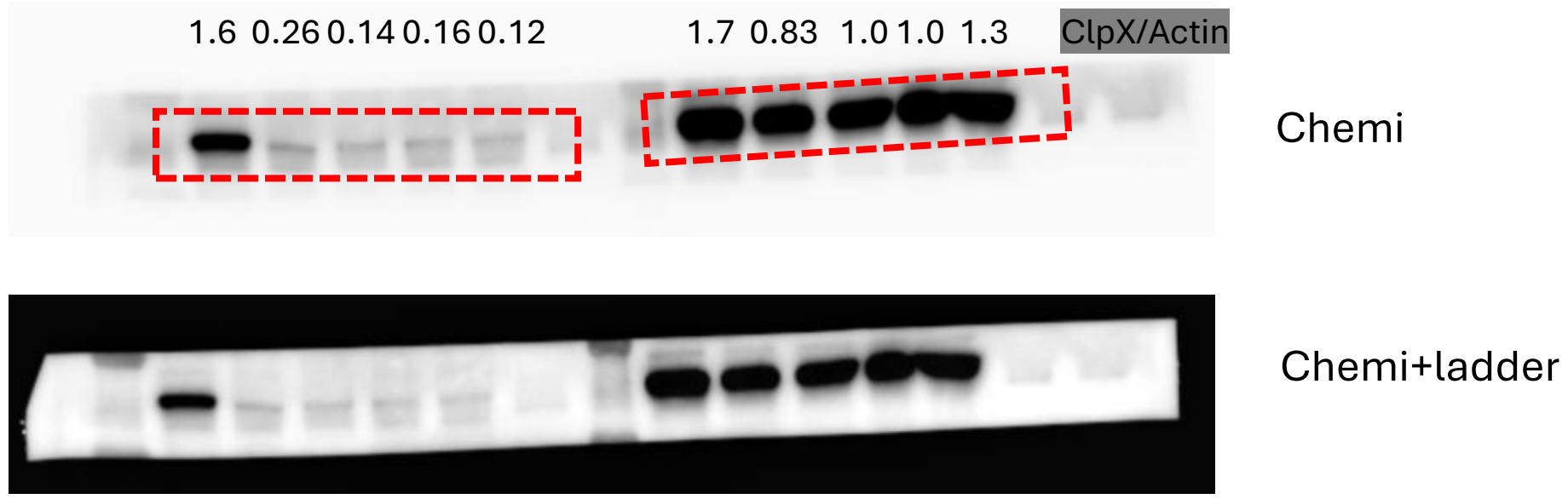

ClpX

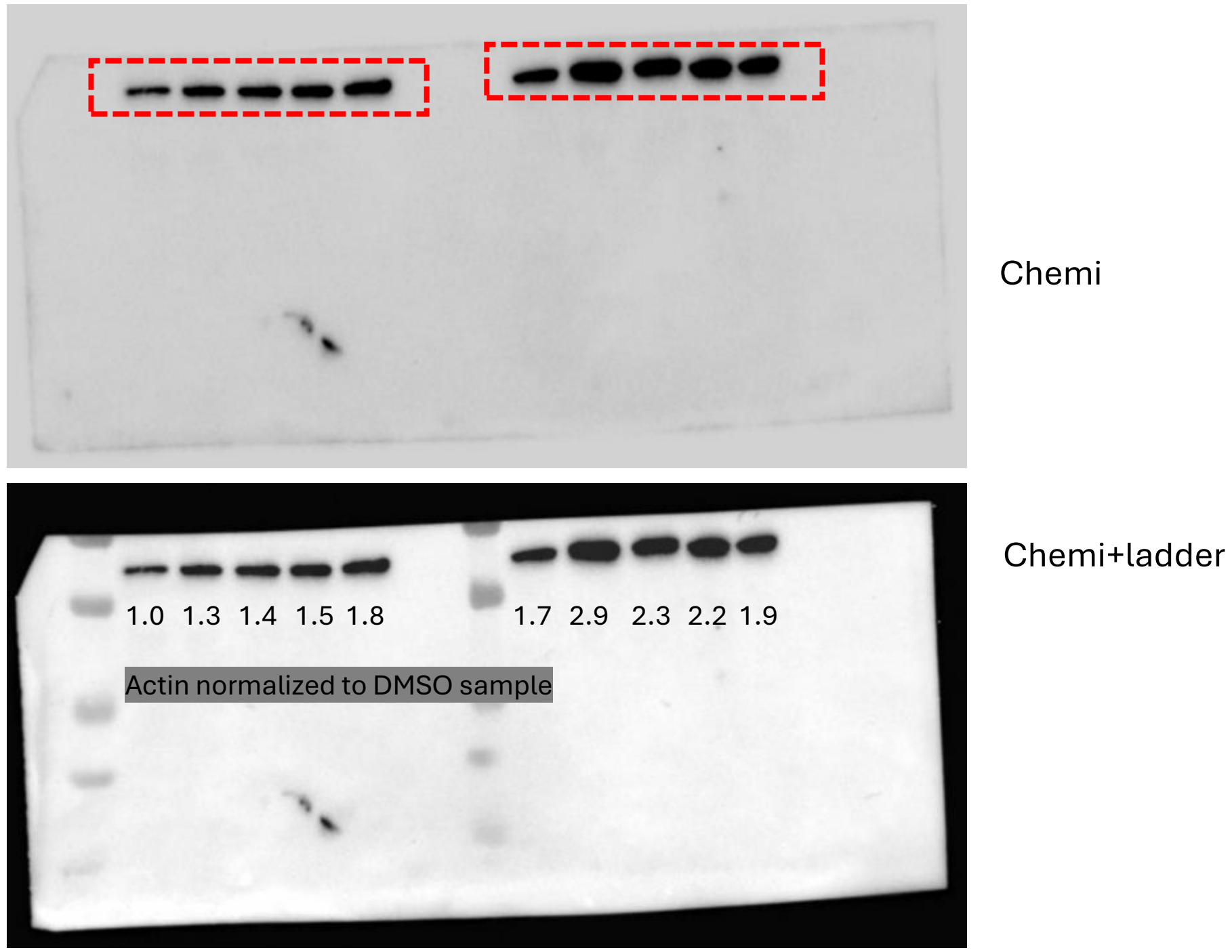

Actin

Supplementary Figure 5C

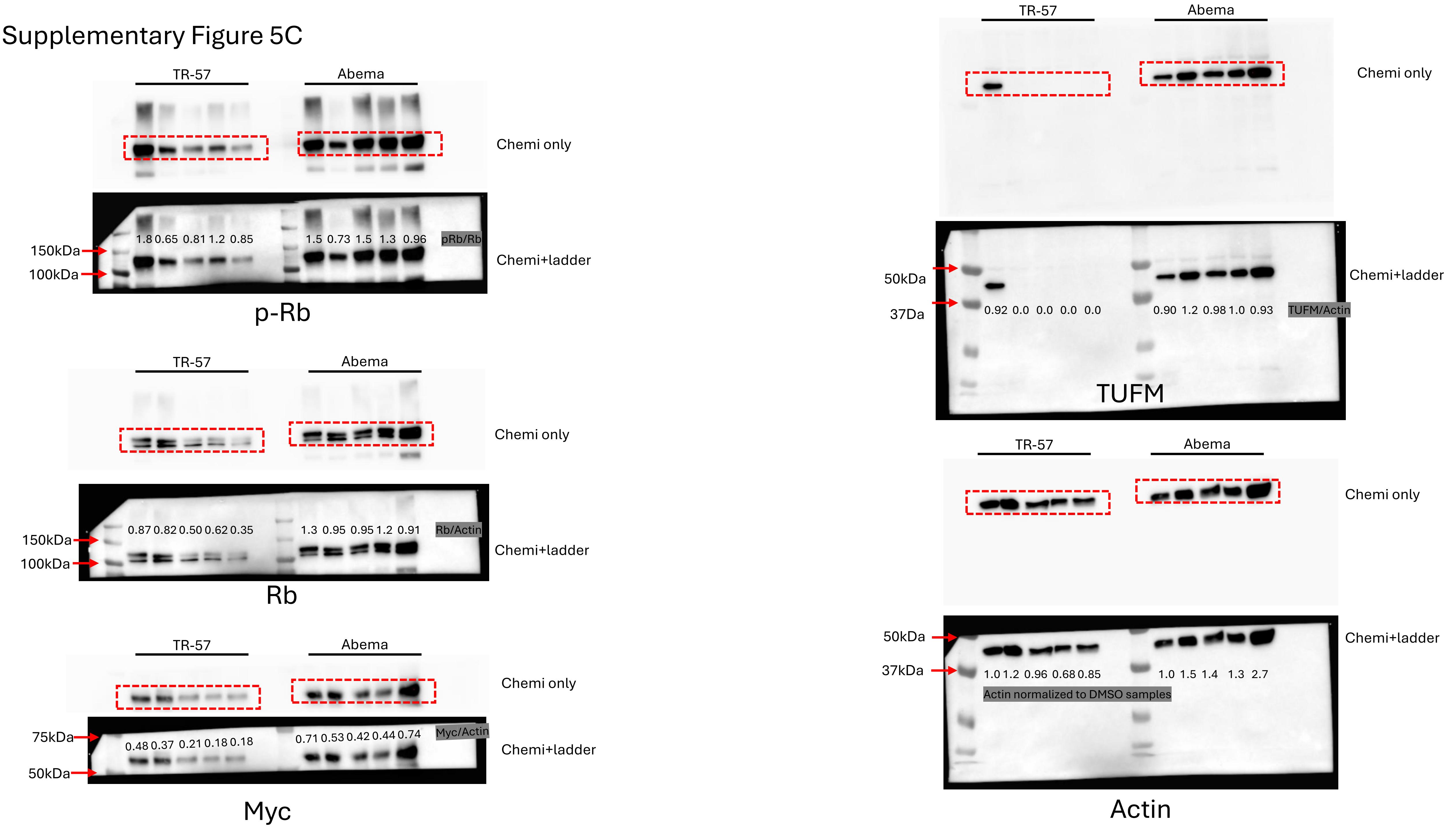

Supplementary Figure 5E

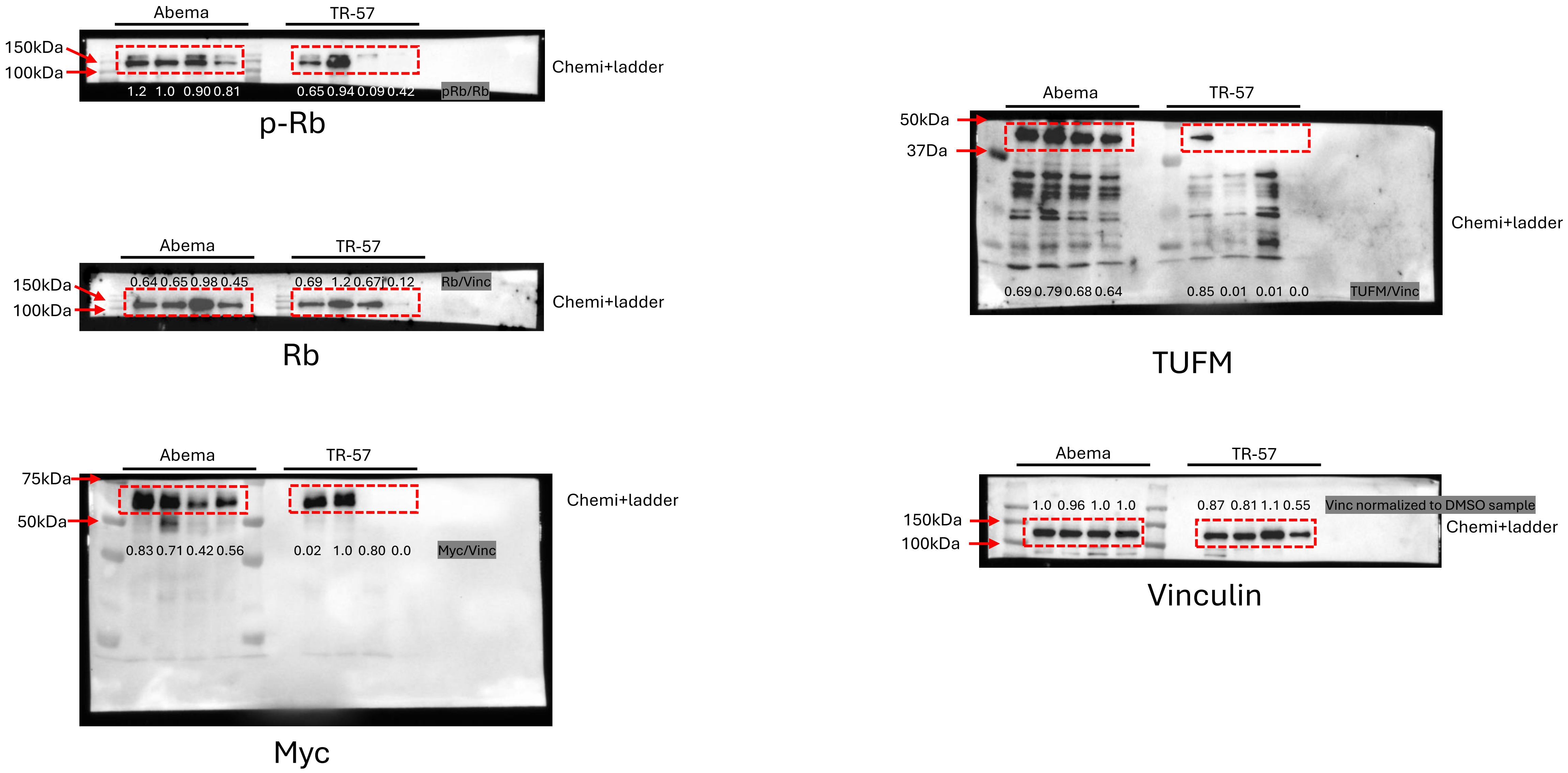

Supplementary Figure 5D

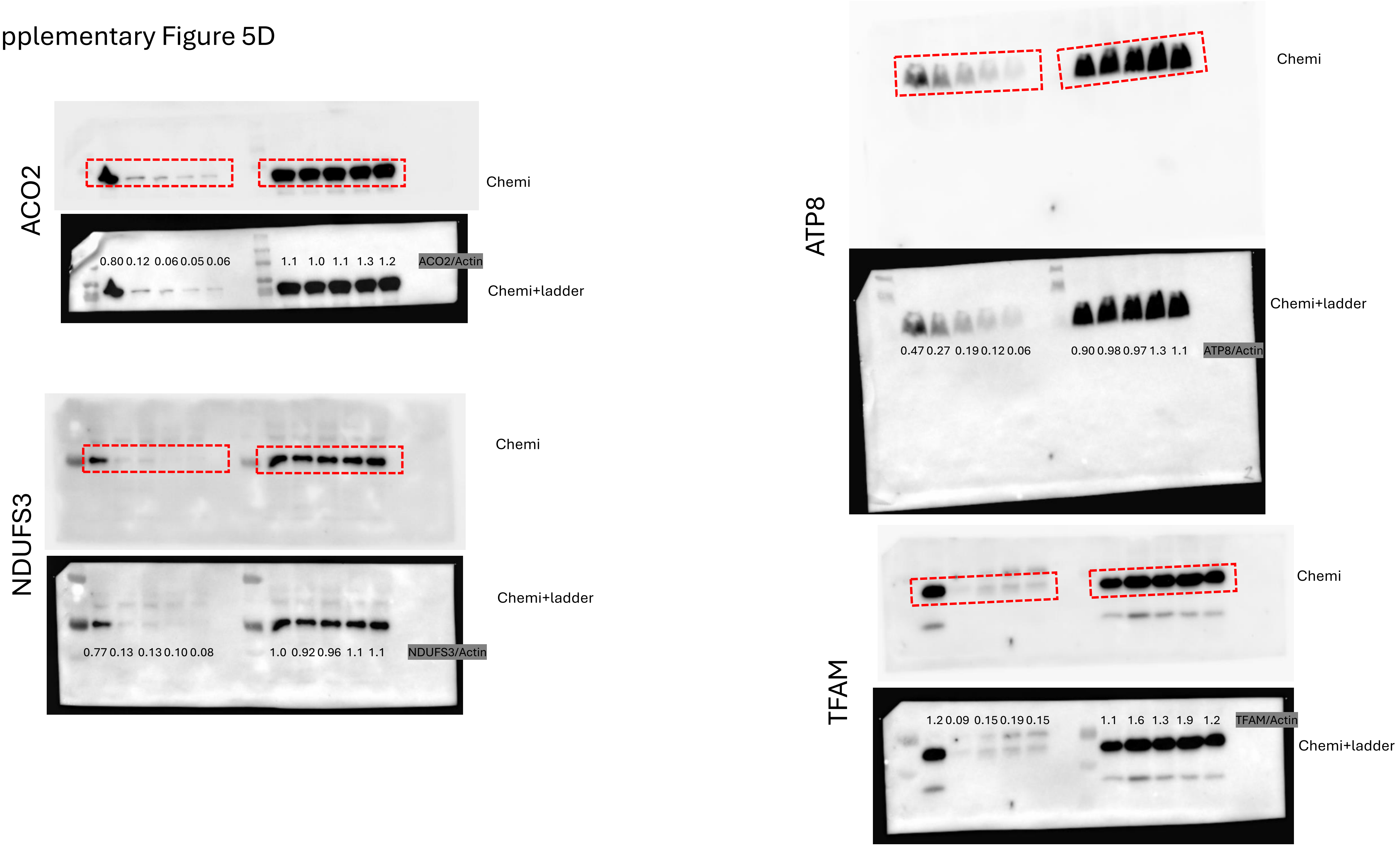

Supplementary Figure 5D continued

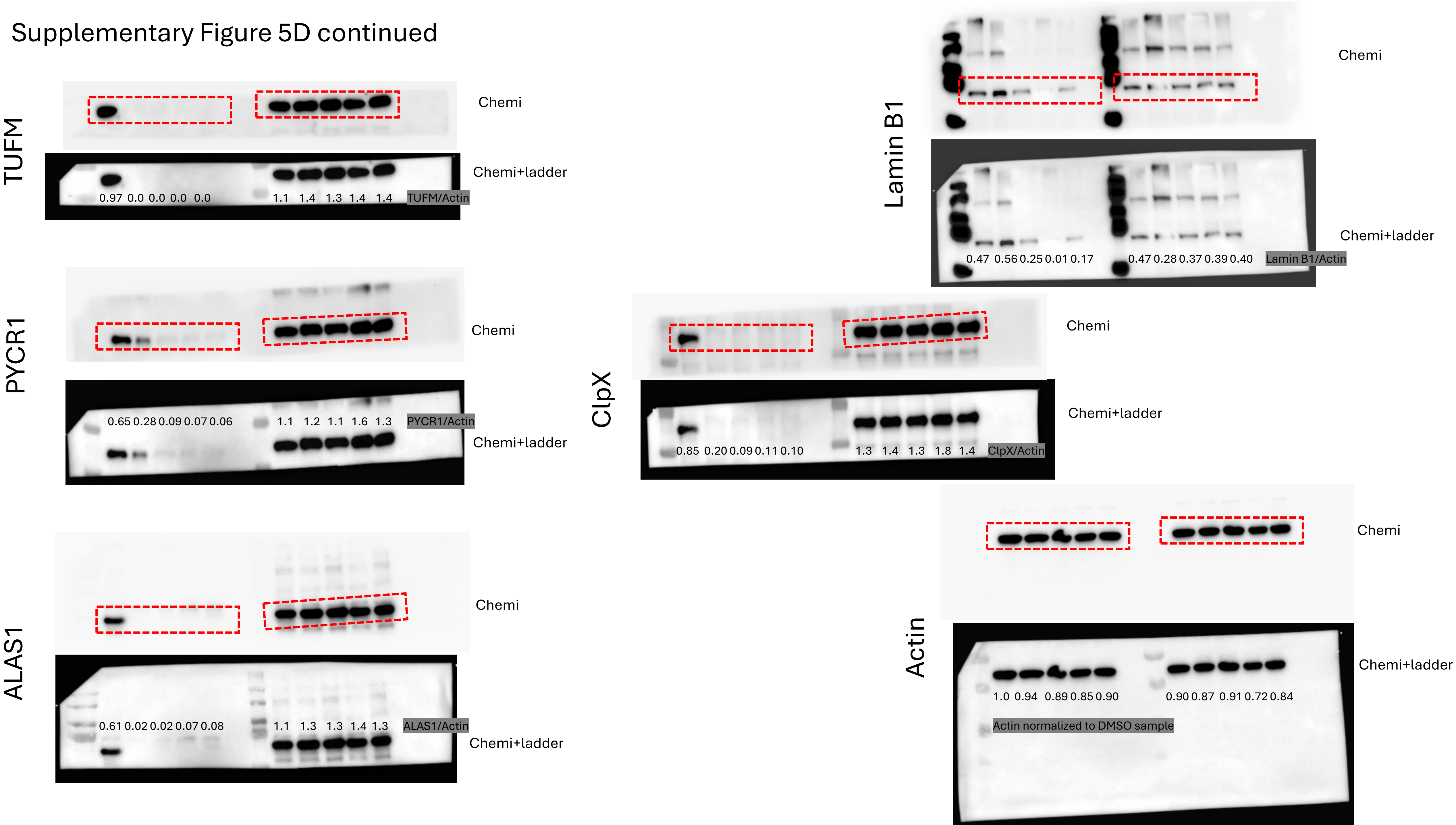

Supplement: Supplementary file 7 [file 41419_2026_9061_MOESM8_ESM.pdf]
