# Supplementary material for: Small molecule activators of the mitochondrial protease ClpP induce senescence in triple-negative breast cancer cells and sensitize cells to the Bcl-2 inhibitor venetoclax
Source: Cell Death Dis. 2026 Jun 29;17(1):803. doi: 10.1038/s41419-026-09061-w (PMC13578376; doi:10.1038/s41419-026-09061-w)
Supplement: Supplementary file 8 [file 41419_2026_9061_MOESM1_ESM.docx]

**Supplementary Figure Legends:**

**Supplementary Figure 1: ClpP Agonists Induce Senescence in TNBC cells**

A) Representative images of quantified β-gal staining data from Figure 1B. B) Representative images of MDA-MB-231 cells from Fig 1C. C) Representative images of SUM159 cells from Fig 1D. D) SUM159 and ClpP KO cells were treated with abemaciclib (48h) or TR-57 for 24-72h and immunoblotting was performed to measure protein expression of senescence markers. Representative images of N=2. E) Immunoblot of GDF15 expression with increasing concentrations of TR-57 (nM) in SUM159 cells. N=2. F) Comparison of dose-response curves from 3 or 30 days of TR-57 treatment. IC_50_ values were plotted for both cell lines in the graph as shown on the right. Representative curves from N=3. G) Immunoblot of TR-57, TR-107, ONC201, abemaciclib, and irinotecan (2μM) demonstrating changes in protein expression of senescence markers. Representative images of N=2. H) Representative immunoblot of ClpP expression in TNBC cell lines presented in this paper. N=2.

**Supplementary Figure 2: ClpP Agonists Deplete Pro- and Anti-Apoptotic Proteins**

A) SUM159 and MDA-MB-231 cells were treated with 0.1% DMSO or 150nM TR-57 for 48h and an immunoblotting array was utilized on cell lysates to visualize proteins known to regulate apoptosis. B) Quantification of SUM159 array. C-D) SUM159 cells and MDA-MB-231 cells respectively were treated with 0.1% DMSO, 100nM staurosporine (S) for 24h, 500nM abemaciclib (A) for 48h, or 150nM TR-57 for 48h. Validation of changes in protein expression from array was performed by immunoblotting. Representative images of N=2-3.

**Supplementary Figure 3: ClpP Agonists Induce S-phase Arrest**

A) Gating scheme for cell cycle analysis in Figure 3. The concentrated population of cells on the left were gated as cells in G1, and on the right as G2/M. The population of cells in the arch above G1 and G2 are EdU+ cells in S phase (S EdU+). The cells in between G1 and G2 populations without EdU staining are also in S phase (S EdU-). B) Representative plots of SUM159, MDA-MB-231 and respective ClpP KO cells treated with 0.1% DMSO or 150nM TR-57 for 24-72h from Figure 3B-C. C) MDA-MB-231 cells were treated with 150nM TR-57, 100nM TR-107, or 10μM ONC201 for 72h. Immunoblotting was performed on cell-cycle proteins. Representative images of N=2.

**Supplementary Figure 4: ClpP Agonists Induce DNA Damage in TNBC cells**

A) MDA-MB-231 and ClpP KO cells were treated with 0.1% DMSO (D) for 48h, 2μM irinotecan (I) for 48h, and 150nM TR-57 for 24-72h. Immunoblotting was performed to detect DNA damage markers. Representative images of N=2. B) MDA-MB-231 cells were treated with 150nM TR-57, 100nM TR-107, 10μM ONC201, 500nM abemaciclib, or 2μM irinotecan for 72h. Immunoblotting was performed on DNA damage markers. Representative images of N=2. C-D) SUM159 cells were treated with the indicated drugs for 48h and immunoblotted as shown. A positive control, indicated by (+ Ctrl), consisted of 250μM H_2_0_2_ for 30 min for all samples except p-Chk1 and Chk1 which was 50μM etoposide for 2h. N=2. E) MDA-MB-231 cells were treated with 0.1% DMSO (D), 1μM Rotenone (R), 150nM TR-57 (TR), and/or 5mM NAC for 48h. Immunoblots of γH2A.X were performed to assess changes in DNA damage. Representative images of N=2. F) MDA-MB-231 cells were treated with 0.1% DMSO, 50μM Teriflunomide (TF), 150nM TR-57 (TR), and/or 30μM Uridine, 100μM Cytidine, and 100μM Thymidine (U+C+T), or 100μM Adenosine and 100μM Guanosine (A+G) for 72h. Representative images of N=2.

**Supplementary Figure 5: TR-57-induced senescence is not reversible in *in vitro* and *in vivo* TNBC models**

A) SUM159 and MDA-MB-231 ClpP KO cells were treated with 0.1% DMSO (D), 500nM abemaciclib, or 150nM TR-57 for 48h before drug washout. Cell count was monitored for 6 days after drug washout. N=3. B) Representative images of β-gal staining from Fig 5B. C) MDA-MB-231 cells were treated with above drugs for 72h before drug washout. Cells were collected every 2 days after washout for up to 6 days. Immunoblotting was performed on proliferation markers and TUFM. Representative images of N=2. D) Representative immunoblots of mitochondrial proteins in MDA-MB-231 and ClpP KO cell lines treated with drug washout scheme. N=2. E) Weights of mice from experiment described in Figure 5F. F) 4T1-Luc cells were treated with 0.1% DMSO or 150nM TR-57 for 24-72h and immunoblotting was performed on proliferation markers and TUFM to demonstrate ClpP activation. Representative images of N=2.

**Supplementary Figure 6: Venetoclax or Olaparib Co-Treatment with ClpP Agonists Increase Cell Death in TNBC Cells**

A-B) Example gating scheme and representative plots for SUM159 and MDA-MB-231 cells respectively. C) SUM159 cells were treated with 0.1% DMSO, 10μM venetoclax (Veneto), and/or 25nM TR-107 for 72h. Annexin V/PI staining was quantified to determine %Apoptotic (Annexin V+) or %Dead (PI+). N=3. D) SUM159 cells were treated with DMSO, 150nM TR-57 and/or 10μM Veneto for 72h. %Dead (PI+) cells were quantified after flow cytometric analysis. N=2.

**Supplementary Method Tables:**

**Table 1: Primers of ClpP sgRNA**

| **Primers for ClpP sgRNA** | **Sequence** |
| --- | --- |
| Forward primer for CLPP1 | TTGGGTGGCCCGGAATATTGGTAG |
| Reverse primer for CLPP1 | AAACCTACCAATATTCCGGGCCAC |
| Forward primer for CLPP2 | TTGGGGGGATGTGGCCCGGAATAT |
| Reverse primer for CLPP2 | AAACATATTCCGGGCCACATCCCC |
| Forward primer for CLPP3 | TTGGGCAGCGGTGCCTGCACGCGA |
| Reverse primer for CLPP3 | AAACTCGCGTGCAGGCACCGCTGC |

**Table 2: Antibodies**

| **Antibody** | **Manufacturer** | **Catalog number** | **Dilution Factor** |
| --- | --- | --- | --- |
| β-Actin | Santa Cruz | SC-47778 | 1000 |
| Vinculin | Santa Cruz | SC-73614 | 1000 |
| Total Rb | Santa Cruz | SC-50 | 1000 |
| Phospho-Rb (Ser807/811) | Cell Signaling | CS-8516 | 1000 |
| Lamin B1 | Cell Signaling | 125865 | 1000 |
| TUFM | Invitrogen | PA5-27511 | 1000 |
| Myc | Cell Signaling | 13987S | 1000 |
| GDF15 | Santa Cruz | SC-377195 | 1000 |
| Bad | Cell Signaling | CS-9292 | 1000 |
| cIAP-1 | Cell Signaling | CS-7065 | 1000 |
| Survivin | Cell Signaling | CS-2808 | 1000 |
| SMAC/Diablo | Cell Signaling | CS-15108 | 1000 |
| Cyclin A2 | Cell Signaling | CS-4656S | 1000 |
| Cyclin D3 | Cell Signaling | 2936 | 1000 |
| Cyclin E1 | Cell Signaling | CS-4129S | 1000 |
| Skp2 | Cell Signaling | 2652 | 1000 |
| p21 | Cell Signaling | CS-2947 | 1000 |
| gH2A.X (S139) | Cell Signaling | 2577S | 1000(IB), 500(IF) |
| p-Chk2 (T68) | Cell Signaling | 2661 | 1000 |
| Chk2 | Cell Signaling | 2662S | 1000 |
| p-S/Q motif | Cell Signaling | 9607S | 100 |
| 53BP1 | Santa Cruz | SC-517281 | 100 |
| Anti-Rabbit Alexa Fluor 568 | Invitrogen | A10042 | 500 |
| Anti-Mouse Alexa Fluor 488 | Invitrogen | A-11001 | 500 |
| p-Chk1 (S345) | Cell Signaling | 2348 | 1000 |
| Chk1 | Cell Signaling | 2360 | 1000 |
| p-ATM (S1981) | Abclonal | AP1030 | 1000 |
| ATM | Cell Signaling | 2873 | 1000 |
| p-ATR (T1989) | Cell signaling | 30632 | 1000 |
| ATR | Cell signaling | 13934 | 1000 |
| Anti-Rabbit IgG (H+L) HRP conjugate | Promega | W4011 | 10000 |
| Anti-Mouse IgG (H+L) HRP conjugate | Promega | W4021 | 10000 |
| ACO2 | Cell Signaling | 6922S | 1000 |
| ATP8 | ProteinTech | 26723-1-AP | 1000 |
| ALAS1 | Abcam | ab154860 | 1000 |
| ClpX | Invitrogen | PA5-79052 | 1000 |
| PYCR1 | Cell Signaling | 47935 | 1000 |
| TFAM | Santa Cruz | sc-376672 | 1000 |
| NDUFS3 | Abcam | ab183733 | 1000 |

**Table 3: Primers for RT q-PCR**

| **Primer target** | **Sequence** |
| --- | --- |
| Forward primer for 18S | AGTCGGAGGTTCGAAGACGAT |
| Reverse primer for 18S | GCGGGTCATGGGAATAACG |
| Forward primer for IL-6 | CCTTCTCCACAAGCGCCTTC |
| Reverse primer for IL-6 | TCTTCTCCTGGGGGTACTGG |
| Forward primer for IL-8 | ACTGAGAGTGATTGAGAGTGGAC |
| Reverse primer for IL-8 | AACCCTCTGCACCCAGTTTTC |
| Forward primer for IL-12 | TCCAGAAGGCCAGACAAACTC |
| Reverse primer for IL-12 | TCCAATGGTAAACAGGCCTCC |
